# Viral genome sequencing to decipher in-hospital SARS-CoV-2 transmission events

**DOI:** 10.1038/s41598-024-56162-7

**Published:** 2024-03-08

**Authors:** Elisabeth Esser, Eva C. Schulte, Alexander Graf, Alexander Karollus, Nicholas H. Smith, Thomas Michler, Stefan Dvoretskii, Angel Angelov, Michael Sonnabend, Silke Peter, Christina Engesser, Aleksandar Radonic, Andrea Thürmer, Max von Kleist, Friedemann Gebhardt, Clarissa Prazeres da Costa, Dirk H. Busch, Maximilian Muenchhoff, Helmut Blum, Oliver T. Keppler, Julien Gagneur, Ulrike Protzer

**Affiliations:** 1 Institute of Virology, School of Medicine & Health, Technical University of Munich/Helmholtz Munich, Munich, Germany; 2 School of Computation, Information and Technology, Technical University of Munich, Garching, Germany; 3 Department of Psychiatry, University Hospital, LMU Munich, Munich, Germany; 4 Institute of Psychiatric Phenomics and Genomics, University Hospital, LMU Munich, Munich, Germany; 5 Department of Psychiatry, University Hospital, Medical Faculty, University of Bonn, Bonn, Germany; 6 Institute of Human Genetics, University Hospital, Medical Faculty, University of Bonn, Bonn, Germany; 7 Laboratory for Functional Genome Analysis, Gene Center, LMU Munich, Munich, Germany; 8 NGS Competence Center, University of Tübingen, Tübingen, Germany; 9 Method development, Research Infrastructure & IT (MFI), Robert-Koch Institute (RKI), Berlin, Germany; 10 Department of Mathematics and Computer Science, Freie Universität (FU) Berlin, Berlin, Germany; 11 Project Groups, Robert-Koch Institute (RKI), Berlin, Germany; 12 Institute for Medical Microbiology, Immunology and Hygiene, School of Medicine, Technical University of Munich, Munich, Germany; 13 German Center for Infection Research (DZIF), Munich Partner Site, Munich, Germany; 14 Max Von Pettenkofer Institute and Gene Center, Virology, National Reference Center for Retroviruses, Faculty of Medicine, LMU Munich, Munich, Germany; 15 Institute of Human Genetics, School of Medicine & Health, Technical University of Munich, Munich, Germany; 16 Computational Health Center, Helmholtz Center Munich, Neuherberg, Germany; 17 Present Address: Institute of Laboratory Medicine, University Hospital, LMU Munich, Munich, Germany

**Keywords:** Viral infection, Epidemiology

## Abstract

The SARS-CoV-2 pandemic has highlighted the need to better define in-hospital transmissions, a need that extends to all other common infectious diseases encountered in clinical settings. To evaluate how whole viral genome sequencing can contribute to deciphering nosocomial SARS-CoV-2 transmission 926 SARS-CoV-2 viral genomes from 622 staff members and patients were collected between February 2020 and January 2021 at a university hospital in Munich, Germany, and analysed along with the place of work, duration of hospital stay, and ward transfers. Bioinformatically defined transmission clusters inferred from viral genome sequencing were compared to those inferred from interview-based contact tracing. An additional dataset collected at the same time at another university hospital in the same city was used to account for multiple independent introductions. Clustering analysis of 619 viral genomes generated 19 clusters ranging from 3 to 31 individuals. Sequencing-based transmission clusters showed little overlap with those based on contact tracing data. The viral genomes were significantly more closely related to each other than comparable genomes collected simultaneously at other hospitals in the same city (n = 829), suggesting nosocomial transmission. Longitudinal sampling from individual patients suggested possible cross-infection events during the hospital stay in 19.2% of individuals (14 of 73 individuals). Clustering analysis of SARS-CoV-2 whole genome sequences can reveal cryptic transmission events missed by classical, interview-based contact tracing, helping to decipher in-hospital transmissions. These results, in line with other studies, advocate for viral genome sequencing as a pathogen transmission surveillance tool in hospitals.

## Introduction

In January 2020, the first infections with severe acute respiratory syndrome coronavirus 2 (SARS-CoV-2) were detected in Germany. Starting with these very first infections, viral sequencing data was used to better understand the disease transmission^1^. With the help of viral sequencing data, it has also been possible to determine the origin and approximate time of the introduction of the virus to e.g. New York City, California, Iceland, or Bavaria^2–5^. Similarly, genomic analysis has been applied to trace local infection chains in hospitals and care facilities^6–8^. SARS-CoV-2 remains an important challenge to healthcare facilities daily, highlighting the importance of defining transmission pathways to improve the safety of patients and healthcare workers alike. The need to better understand transmission chains advocates for the use of viral genome sequencing. However, so far it is not well-understood how state-of-the-art viral genome sequencing combined with bioinformatics approaches for transmission tracing compares to conventional, interview-based, contact tracing in the setting of healthcare institutions. This extends far beyond the current SARS-CoV-2 pandemic to all common infectious diseases encountered in clinical and hospital settings.

This analysis aimed to understand in-hospital transmission clusters of SARS-CoV-2 at the university medical center of the Technical University of Munich (TUM) (Klinikum rechts der Isar), in Munich, Germany, by SARS-CoV-2 whole viral genome sequencing in combination with two bioinformatically defined clustering approaches compared to interview-based contact tracing.

## Methods

Viral whole genome sequences were obtained from residual diagnostic material positive for SARS-CoV-2 by PCR. Samples were collected at Klinikum rechts der Isar in Munich, Germany, from February 3, 2020, to January 10, 2021 (“TUM samples”, Suppl Fig. 1, cf. Suppl Methods). *CleanPlex*®^9^ or *Artic*^10^ SARS-CoV-2 sequencing panels were used for library preparation. Sequencing was performed on *Illumina* platforms for a total of 926 samples from 622 probands at three sequencing sites across Germany (cf. Suppl Methods). A proband is defined as any SARS-CoV-2-positive individual included in the study.

*Bwa-mem*^11^ and *freebayes*^12^ were used to align sequences to the SARS-CoV-2 reference genome (NC 045512.2) and to call variants. After quality control, 619 samples of 475 individuals (320 patients, 155 staff members) were included in the analysis (cf. Suppl Methods). Only sufficiently covered positions of the reference genome were used in variant analyses (coverage larger than 10 reads). Sites with missing information (coverage of 10 reads or less) were not used in any of the computational analyses but were displayed in the figures for ease of interpretation.

An additional dataset of local viral sequences from 16 hospitals and emergency departments across Munich other than the Klinikum rechts der Isar, collected by the LMU, served as negative controls. The inclusion of all viral sequences available at the time in Munich served as proxy for the viral strains circulating at the time in the area from which the hospital patients stem. It was utilized as a negative control to assess evidence of transmission taking place in the hospital. At the time, the same containment measures applied to the entire area covered by all hospitals analysed and there were no restrictions regarding a patient’s choice of hospital, reducing the probability of localized transmission chains. This additional dataset consisted of 1,156 samples (“LMU samples”, cf. Suppl Methods). Viral whole genome sequencing was performed using the *Artic* protocol^10^ on the *Illumina* platform at the Gene Center of the LMU. Alignment, variant calling, and quality control were performed as outlined above, resulting in 957 samples from 854 individuals in the analysis (cf. Suppl Methods).

**Figure 1:**
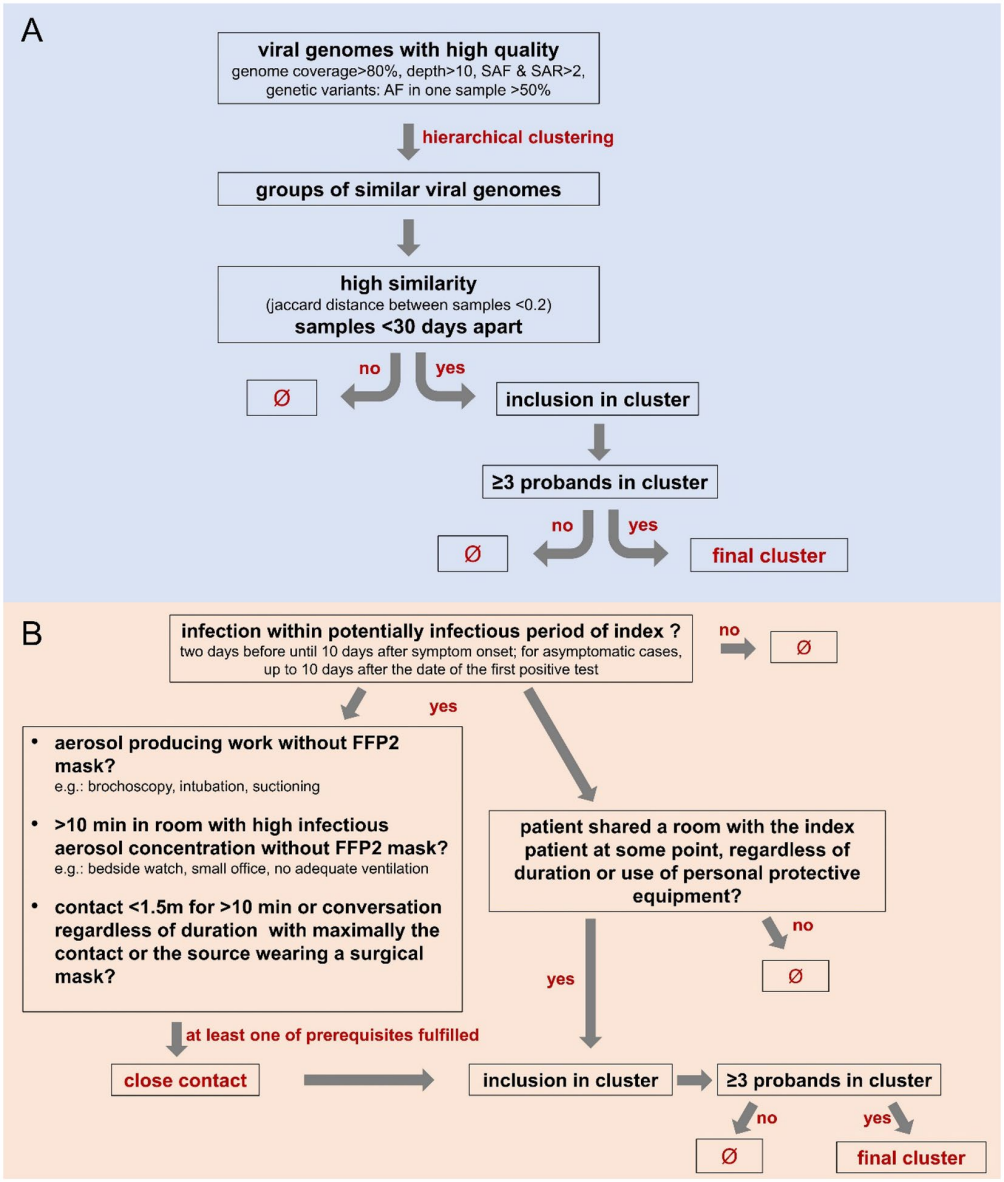
Algorithms used to determine in-hospital transmission clusters (**A**) as part of the viral genome clustering and by (**B**) interview-based patient clustering. Individuals were included in a viral genome cluster when they had high similarity to the other samples in the clusters and were in the timeframe of 30 days in comparison to the other samples All clusters were obligated to contain at least three individuals. (SAF = Number of alternate observations on the forward strand, SAR = Number of alternate observations on the reverse strand). Individuals were included in an interview-based cluster when the infection occurred within the potentially infectious period of the index patient and there was reported close contact or the patient shared a room with the index patient, regardless of duration or use of personal equipment.

Hierarchical clustering was performed in *RStudio* (version 4.1.1) using as dissimilarity the pairwise Jaccard distances computed on shared covered bases and the agglomeration method “*complete*”. The resulting clusters were visualised as phylogenetic trees, which were cut, according to a *Nextclade* ^13^-derived algorithm, to put similar samples into groups (cf. Suppl Methods). These groups were filtered for high similarity (Jaccard distance (JD) between samples < 0.2), time of sample collection (< 30 days apart, an estimate based on incubation and infectious period), number of probands included (≥ 3) (cf. Suppl Methods). Genetic variants were only included in the analysis if they occurred in at least one sample with an allele frequency higher than 50%. (Fig. 1A).

Additional metadata for the TUM samples included work location (staff) and duration of hospital stay as well as wards and ward changes (patients). The institutional review board at the Technical University of Munich (100/22-S-KH) approved the study. As part of routine procedures, interview-based contact tracing was performed by a physician specialising in clinical hygiene whenever a transmission cluster was suspected clinically. Individuals were included in an interview-based transmission cluster when either a close contact was reported, or when a patient had shared a room with the index patient at some point (Fig. 1B, cf. Suppl Methods). A cluster was defined when at least three infections were connected as described above. This interview-based procedure focused on the cause of primary infections and, therefore, was not designed to detect cross-infections.

To validate in-hospital transmissions, for a representative period from September 2020 to January 2021, we calculated the number of virus genomes with closely related genomes (JD < 0.2) for each genome in the TUM dataset (n = 334). Using Monte Carlo simulations, this number was then compared to the same measure for an equal number of “negative” control genomes (LMU dataset, n = 681) from other healthcare facilities across Munich from the same time period (cf. Suppl Methods). A similar approach was used to confirm true transmission clusters ruling out multiple simultaneous introductions. For each Jaccard-based cluster, the number of closely related viral genomes was calculated in the LMU dataset. If this number was smaller than the number of samples in the Jaccard-based cluster in 95% of the results, the cluster was deemed likely to be an in-hospital transmission cluster (cf. Suppl Methods).

Complementary to our Jaccard-based clustering, we performed a phylogenetic analysis with *Nextstrain* (release V13)^13,14^ with default settings, without the ‘diagnostics’ filter as it removed too many samples. Consensus sequences of all TUM and LMU samples were included as well as consensus sequences of all additional samples (n = 61) from Munich available on *GISAID*^15^ (EPI_SET_231229gn; https://doi.org/10.55876/gis8.231229gn). The phylogenetic tree was obtained using the *Nextstrain* pipeline^14^, restricted to samples passing *Nextstrain* quality control filters (n = 1322).

In order to statistically assess in-hospital transmissions using *Nextstrain*-based phylogenetic trees, we built another tree in which only the latest sample of probands with multiple samples was used (n = 1249 samples in total). Next, the distance between each TUM sample and any of its closest TUM samples of the same month in the tree was computed. This distance represents the number of mutations separating these samples according to the phylogeny inferred by the *Nextstrain* pipeline. For every month, we then considered the average across all TUM samples of these distances to their closest TUM sample. The statistical significance of these averages was assessed by permutation testing. Specifically, the same statistic was computed 500 times upon random permutation of the TUM labels among all sample origin labels of the same month, and p-values were estimated as described by Phipson and Smyth^16^. For comparability, this analysis was restricted to the months (September 2020 to January 2021) in which the TUM samples were sequenced with the *Artic* protocol (cf. Suppl Methods) which is the protocol used for all other Munich samples. Moreover, we discarded September 2020 as it had too few samples (n = 47).

### Ethics approval

The institutional review board at the Technical University of Munich (100/22-S-KH) approved the study and allowed the acquisition of a minimal dataset without informed consent.

### Consent to participate

All methods were performed in accordance with relevant guidelines and regulations.

## Results

### Clustering

Jaccard-based clustering of 619 high-quality viral genomes (Suppl Fig. 23) from 475 probands generated 19 clusters ranging in size from three to 31 individuals (mean ± SD: 9.5 ± 8.1). Of the 475 probands, 179 were included in at least one cluster, six had samples clustering in more than one cluster (Suppl Fig. 2 to 20). In the early months, which represent the first wave of SARS-CoV-2 infections (January 2020 to June 2020), the viral strains show low genetic diversity, making it difficult to distinguish between actual transmission and incidental genetic similarity. Furthermore, there was no interview-based contact tracing data available for this period. For these reasons the period from February 2020 to June 2020 was not considered in further analysis.

**Figure 2:**
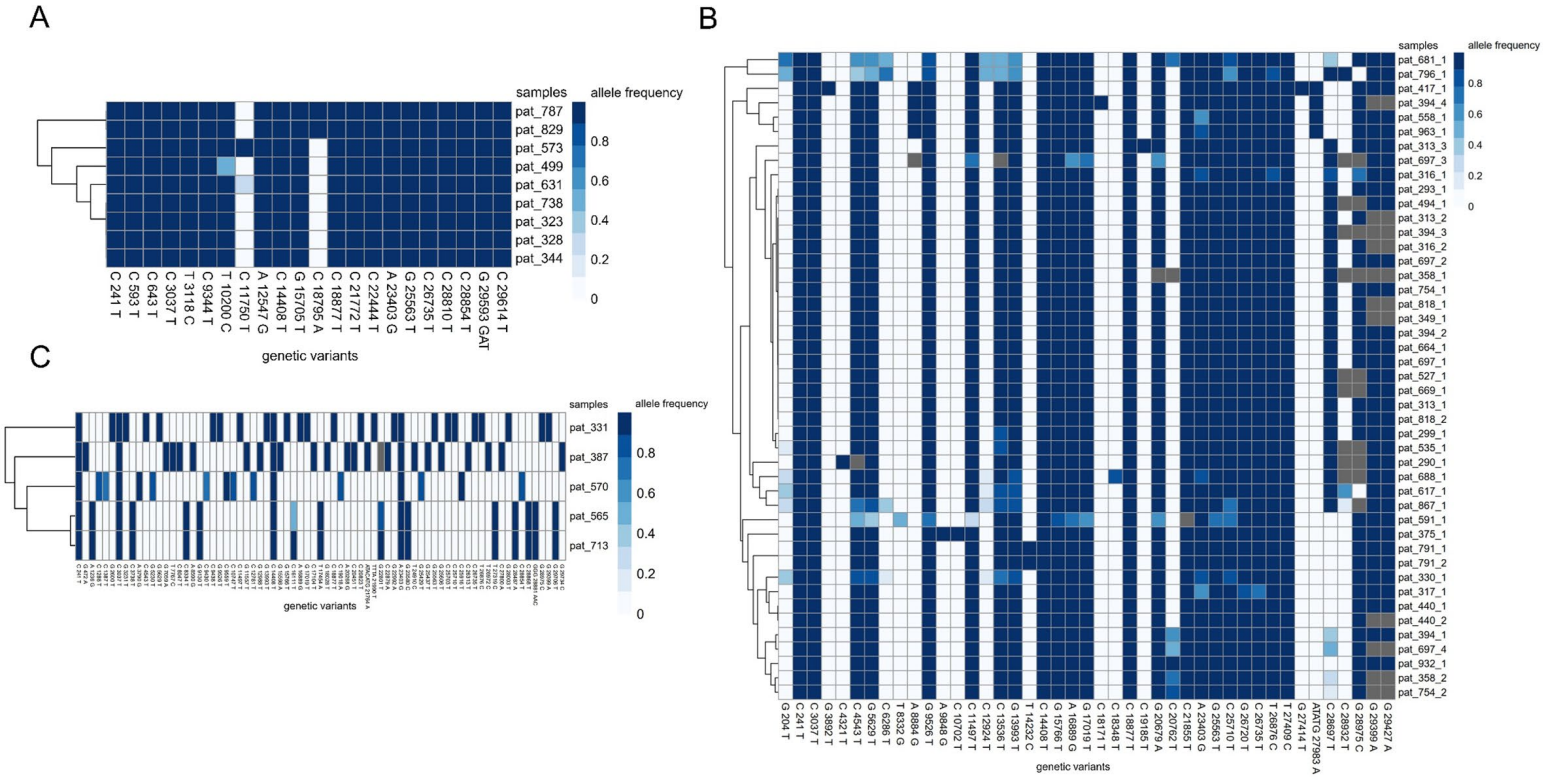
Examples of transmission clusters. (**A**) Viral genome clustering confidently identifies clusters of individuals working in a non-health-care-related sector that very likely infected each other as a proof-of-principle (Cluster M). (**B**) Example of a viral genome cluster (Cluster H) of intra-hospital infections. This largest of 19 clusters includes 45 samples of 31 individuals. (**C**) Example of an interview-based contact tracing cluster showing overall lower genetic relatedness. The color gradient depicts allele frequencies, grey coloring denotes that the base call did not pass the quality control filters for a given variant. X-axes display genetic variants with allele frequencies > 50% in at least one sample when compared to the reference genome (NC 045,512.2) Each row denotes one sample included in the cluster.

As proof of principle, Jaccard-based clustering confidently identified a cluster of patients with a high professional risk of infection from working in a non-health-care-related sector that very likely infected each other (Fig. 2A, Cluster M).

**Figure 3:**
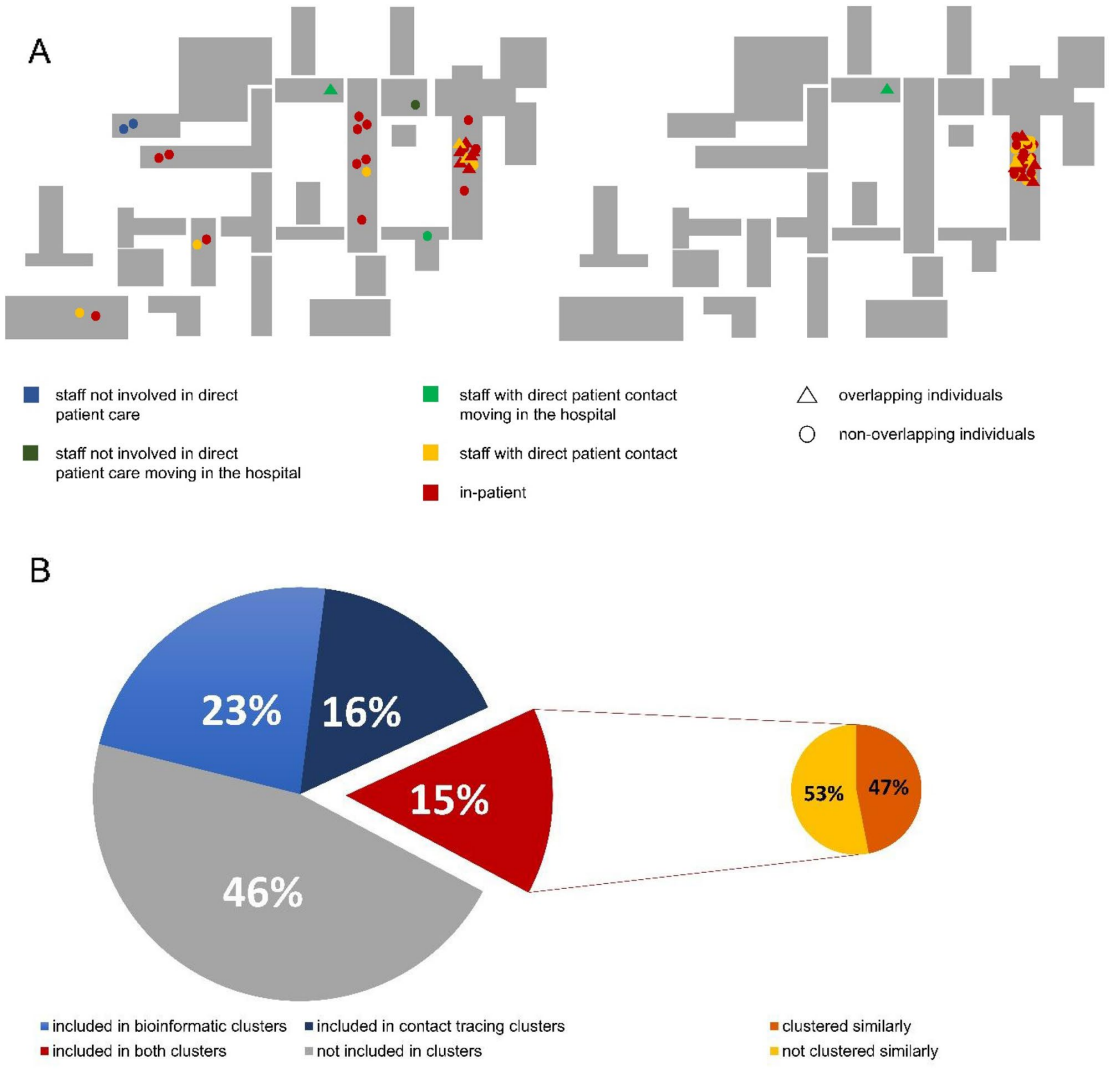
Example of the spatial distribution of infections over the hospital and distribution of individuals included in cluster analysis (**A**) Comparison of (left) the biggest viral genome cluster to (right) interview-based contact tracing, which have an overlap of 9 individuals (triangle), which were included in this cluster by both approaches. The viral genome cluster spans a significantly larger portion of the hospital, while the contact tracing cluster mostly includes individuals from one part of the hospital. (**B**) For the timeframe with interview-based contact tracing data available, 334 individuals were included in the clustering analysis. 103 probands formed part of an interview-based contact tracing cluster and 126 part of a viral genome cluster. There is an overlap of 50 probands (15%) which were included in clusters by both approaches. Over half of those were clustered together differently by the two approaches.

The largest Jaccard-based cluster included eleven staff members and 20 patients (Fig. 2B, Cluster H). Of the staff members, three worked on the same non-COVID ward, three were from services that move within the hospital, two from a service area, which is not involved in direct patient care, and three from non-adjacent wards. In order to understand how the Jaccard-based clusters relate to the clinical situation, a retrospective case-by-case review of the clinical data (date and reason of admission, ward movements, patient histories) was performed. Based on these chart reviews, the likely route of infection was established. Of the 20 patients, seven were deemed to have hospital-acquired infections, and five were deemed very likely to have hospital-acquired infections. In these cases, the time between their admission and the first positive PCR-result was seven to 15 days, suggesting that the infection occurred after admission. The remaining eight patients had a cryptic path of transmission. Only three staff members and nine patients were associated with the same non-COVID ward, suggesting a cluster spanning more than just one ward. This cluster was also identified by interview-based contact tracing with an overlap of nine individuals, which were included in this cluster by both approaches. The much greater distance of within-hospital dissemination in this cluster suggested by the viral genomes when compared to interview-based contact tracing data is also evident when plotting the work and treatment locations of all probands in the cluster on a schematic representation of the hospital (Fig. 3A).

The interview-based contact tracing analysis spanned October 2020 to January 2021, resulted in 12 clusters (range: 3–51 individuals; mean ± SD: 17.9 ± 18.5) and included 209 individuals. 103 hereof also had high-quality viral whole genome sequencing data and were included in the bioinformatic analysis.

Viral genome sequencing suggests large heterogeneity between the clusters identified in the same dataset using the two different approaches. Many of the viral samples in interview-based contact tracing clusters are not closely genetically related. Only a mean of 22.1% of the included samples has a close relationship to other samples in the cluster (JD < 0.2), making direct transmission between most of the samples included in the clusters identified by interview-based contact tracing unlikely (Fig. 2C). In most of the Jaccard-based clusters, a few probands (ranging from 6.9% to 33.3%, mean ± SD: 22.5% ± 9.3%) were also clustered together by contact tracing. There is an overlap of 50 probands which were included in interview-based contact tracing clusters (n = 103 probands) and Jaccard-based clusters (n = 126 probands). However, in many instances, probands were assigned to different clusters by the different strategies. 61.1% of probands in the Jaccard-based clusters were not found in any of the interview-based contact tracing clusters. (Fig. 3B).

### Validation of in-hospital transmissions

To exclude the possibility that the Jaccard-based clusters are simply due to multiple independent introductions into the hospital setting at times of high local virus circulation, we compared the genetic relatedness in our dataset to viral genome sequencing data from the same time period collected by a second large university hospital in Munich (LMU samples). For the last virus sample collected from each individual in the TUM dataset, the average number of individuals with a closely related virus genotype (JD < 0.2) was determined (T_k_ = 7.96). Randomly drawing an equal number (n = 334) of viral genomes from the LMU samples (n = 681) showed that the TUM samples were significantly more closely related to each other than to the LMU samples (T_k_^LMU^ = 1.09–2.00) (Monte Carlo, 1000 permutations, *p* = 0.001, Suppl Fig. 21A&B). Whether the first or the last collected viral genome of an individual was used, made no difference in this analysis (Suppl Fig. 21C).

The analysis, which put the Jaccard-based clusters in context of random draws from the LMU dataset, resulted in the confirmation of eight true transmission clusters (clusters F, G, H, I, J, L, M and S)out of 14 clusters in the observed time frame (clusters F-S(cf. Figure 2 and Suppl Fig. 7 to 20)). It is still possible that in-hospital transmissions took place in the other six clusters, but since the prevalence of closely related samples outside the hospital was high in these cases, multiple independent introductions into the hospital setting cannot be ruled out.

### Phylogenetic analysis with Nextstrain

**Figure 4:**
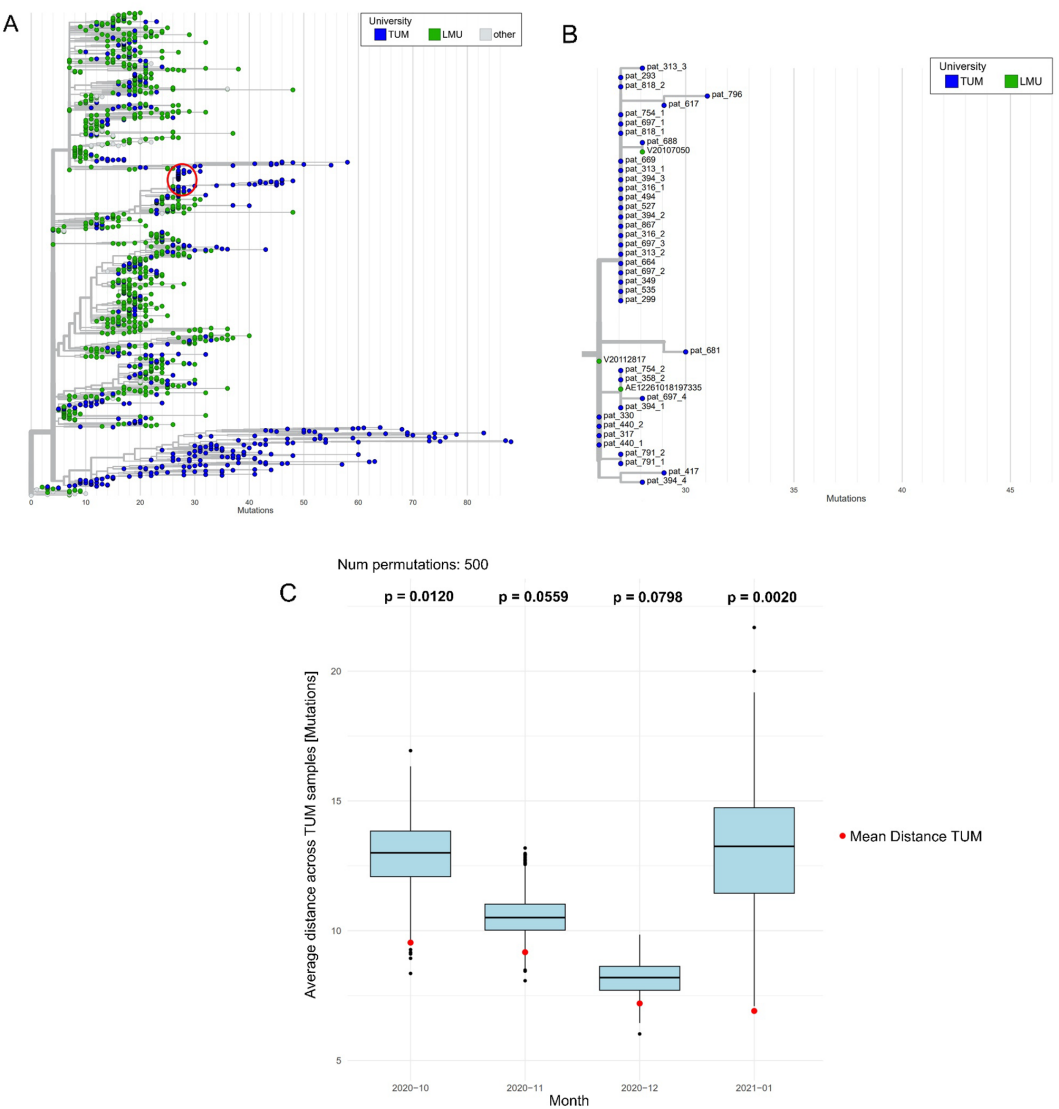
Phylogenetic Analysis with Nextstrain. (**A**) Phylogenetic tree created with Nextstrain, containing all samples from Munich during the observed period (from February 3, 2020, to January 10,2021). The red circle depicts the Jaccard-based cluster H as displayed in panel B. (**B**) Example of the visual representation of the Jaccard-based cluster H in the phylogenetic tree obtained with Nextstrain. 38 of the 45 samples in the cluster were included in the tree. (**C**) Results of permutation tests for a representative period from October 2020 to January 2021. Only the last sample of each individual was used (n = 1249).

Complementary to the Jaccard-based clustering, we also considered an approach based on *Nextstrain* phylogenetic trees. We first created a phylogenetic tree using all viral sequences from Munich (Fig. 4A). We compared the Jaccard-based clusters to the *Nextstrain* phylogenetic tree focusing on the period from October 2020 to January 2021 because there were at least 50 samples in each of these months in Munich and because for these months, all the samples were sequenced using the *Artic* protocol. Of the 157 TUM samples in the *Artic* sample clusters (F to S), 118 passed the *Nextstrain* quality filters and were therefore included in the phylogenetic tree. Qualitatively, we found that samples of the Jaccard-based clusters were typically close to each other in the *Nextstrain* tree. Figure 4B exemplifies the comparison between Jaccard-based clustering and *Nextstrain* phylogeny for Cluster H. A comparison between all Jaccard-based clusters and the *Nextstrain* phylogenetic tree can be found in the supplement (Suppl Fig. 7C to 20C). This investigation showed that the phylogenetic *Nextstrain* tree and the Jaccard-based clustering gave qualitatively similar results.

We then statistically assessed in-hospital transmission using *Nextstrain-*based phylogenetic inference. To this end, we built another tree in which we kept a single sample per longitudinal course to discard close phylogenetic relationships between samples from the same patients. We selected the latest sample of each longitudinal course to enrich the sensitivity to detect in-hospital transmission. In this tree, the distance between any TUM sample to its closest TUM sample was significantly closer on average than by chance for every assessable month (Fig. 4C). This analysis further provides evidence for in-hospital instead of community-based transmission.

### Longitudinal samples

**Figure 5:**
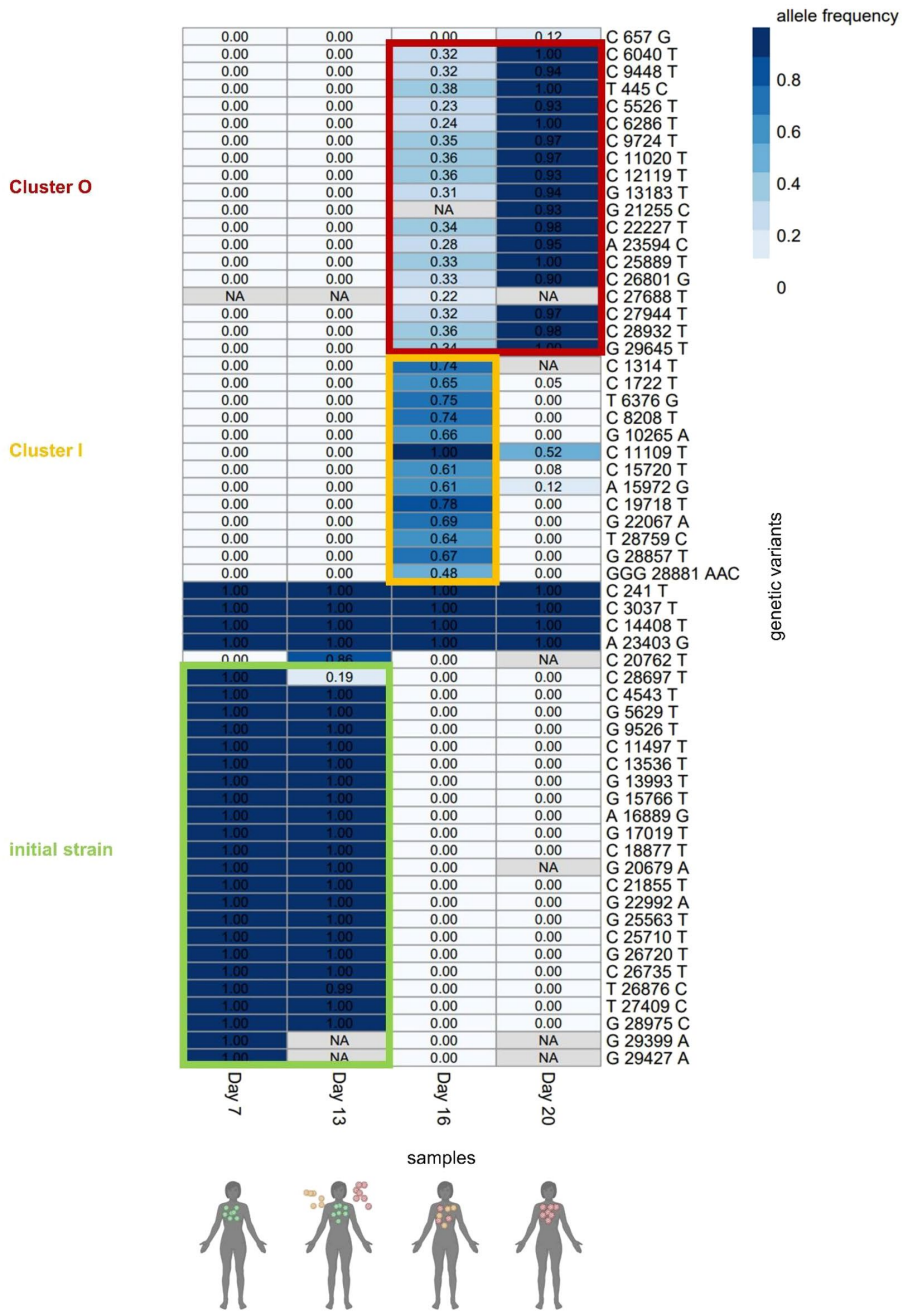
Example of a complete change of virus strain in one patient representing a possible cross-infection. The individual likely presented to hospital with one strain (“initial strain”, outlined in green), then came in contact with two other virus strains, which are both visible in the third sample (outlined in yellow (“Cluster I”) and red (“Cluster O”), and one of the strains (“Cluster O”) remained in the end (last sample from day 20). The “Cluster I” and “Cluster O” virus strains, represent two clusters that were circulating in the hospital at that time, as evidenced by their presence in additional in-hospital clusters identified by viral genome sequencing. The patient was not immunocompromised.

The existence of numerous transmission clusters in the hospital also raises the possibility of cross-infections. Analysis of SARS-CoV-2 whole genome sequencing data from 73 individuals, for whom longitudinal samples were available (average length of longitudinal course ± SD: 11.1 ± 7.4 days; average samples per individual ± SD: 2.7 ± 1.0), revealed patterns of complete change in virus strain during the longitudinal observational period in 14 individuals (19.2%) (Fig. 5). Since virus strain changes occurred within short time spans (mean ± SD: 5.2 ± 3.7 days) and all at once instead of successively, this suggests cross-infection events during the hospital stay rather than in-host viral evolution. In line with this hypothesis, these individuals also appeared in more than one Jaccard-based cluster during their hospital stay. A mix-up of samples cannot fully be excluded, but since most patients were in close proximity to other individuals with the virus strain they eventually acquired, a mix-up is less likely.

## Discussion

Clustering analysis of 619 viral genomes from 475 patients or staff members of a Munich university hospital allowed to define 19 transmission clusters. Viral genomes of TUM were significantly more closely related to each other than to a comparable set of 829 genomes collected simultaneously across the same city, strongly suggesting nosocomial transmission. Comparing these bioinformatically derived, sequencing-based transmission clusters with interview-based contact tracing, however, showed little overlap. Interestingly, longitudinal sampling from individual patients suggested possible cross-infection events during the hospital stay in a significant proportion of the individuals.

Our analysis recapitulates recent findings^6–8,17–19^ that demonstrate the utility of viral genome sequencing in the identification of transmission clusters within healthcare institutions and beyond and identified divergent clusters between viral genome sequencing and interview-based contact tracing. We found that viral genome clusters derived using two different computational approaches tended to be smaller, more closely related genetically and to be spanning spatially larger portions of the hospital. Similarly, for example, a study using SARS-CoV-2 whole genome sequencing analyses in a tertiary referral hospital in Madrid, Spain, showed that the introduction of five different SARS-CoV-2 strains was responsible for what was assumed to be a homogeneous outbreak due to a single transmission chain by interview-based contact tracing^17^. Also, the addition of local viral genome sequencing data covering the same time period from outside the hospital resulted in ruling out the involvement of two cases in the outbreak, due to the high probability of community-acquired infections^17^. Czech-Sioli et al.^8^, in investigating 284 samples in their analysis, came to similar conclusions that temporally preceding index cases and transmission routes can be missed when using only interview-based contact tracing. Through alignment with GISAID data^15^, they also showed that placing sequences in a local context is essential to distinguish independent entries from in-hospital transmission. Additionally, as interview-based contact tracing cannot identify cross-infections, the efficacy of containment procedures cannot be assessed.

The bioinformatic analysis of sequencing data provides information of transmission pathways that were not previously suspected. This includes, for example, staff members from service areas, which are not involved in direct patient care, for whom no connection to transmission clusters was expected. Further, by providing largely unbiased, depersonalized information, transmission chain tracing using viral genome sequencing will eliminate the (perceived) denunciation involved in personal contact tracing while at the same time providing more accurate results.

Virus genome sequencing also harbours the potential to better understand cryptic transmission events at micro-scale. Several studies, describing patterns of within-host diversity, found evidence of co-infections^20–22^ and that co-infection with certain strains might be driven by infection from two different sources of infection^20,21^. This could be similar to our observations where the predominant virus strain in almost 20% of individuals for whom this data was available changed during the hospital stay. While we cannot fully exclude the possibility of sample mix-ups or cross-contaminations, this surprisingly high number of individuals with more than one viral strain during their hospital treatment highlights the continued importance of individual isolation measures in SARS-CoV-2-infected individuals to limit in-hospital viral persistence and spread.

While likely more precise in identifying in-hospital clusters of transmission for SARS-CoV-2, there still are important limitations to the viral genome sequencing approach that challenge its widespread use. It is not always possible to obtain high-quality genomes with quick turn-around times, due to, for instance, low viral load, technical limitations or high costs. This can result in incomplete datasets and, consequently, transmission chains. Also, the necessary technical and computational resources are often only available at larger-scale academic institutions, hampering its widespread implementation in quotidian clinical practice. Lastly, genetic tracing is challenging for newly emerging pathogens with low genetic diversity, as illustrated by our data during the first wave of SARS-CoV-2 infections (January 2020 to June 2020), where it is difficult to distinguish between actual transmission and incidental genetic similarity.

In our experience, interview-based contact tracing alone is not sufficient to fully map transmission pathways and clusters in a large university hospital. Complementation with viral genome sequencing data proofed very beneficial, especially, by highlighting in-hospital transmission chains that were spatially more expansive than expected. This information is of paramount importance, however, to efficiently contain transmission chains.

While the SARS-CoV-2 pandemic provided an impetus to the implementation of viral genome sequencing in clinical practice, the same advantages will also apply to transmission tracing of nearly all other pathogens and metagenomic approaches can further broaden the scope of pathogens detected.

### Supplementary Information

Supplementary Information.

## Data Availability

All consensus sequences, that met the needed quality requirements, were uploaded to the GISAID repository, https://gisaid.org/, with the according metadata. Genome sequences and associated metadata on GISAID is accessible at https://doi.org/10.55876/gis8.231229gn. The GISAID supplemental table can be found in the supplementary material.

## References

[1] Wölfel R (2020). Virological assessment of hospitalized patients with COVID-2019. Nature.

[2] Gonzalez-Reiche AS (2020). Introductions and early spread of SARS-CoV-2 in the New York City area. Science.

[3] Deng X (2020). Genomic surveillance reveals multiple introductions of SARS-CoV-2 into Northern California. Science.

[4] Gudbjartsson DF (2020). Spread of SARS-CoV-2 in the icelandic population. N. Engl. J. Med..

[5] Muenchhoff M (2021). Genomic epidemiology reveals multiple introductions of SARS-CoV-2 followed by community and nosocomial spread, Germany, February to May 2020. Euro Surveill..

[6] Meredith LW (2020). Rapid implementation of SARS-CoV-2 sequencing to investigate cases of health-care associated COVID-19: A prospective genomic surveillance study. Lancet Infect. Dis..

[7] Lucey M (2021). Whole-genome sequencing to track SARS-CoV-2 transmission in nosocomial outbreaks. Clin. Infect. Dis..

[8] Czech-Sioli M (2022). Integration of sequencing and epidemiological data for surveillance of SARS-CoV-2 infections in a tertiary-care hospital. Clin. Infect. Dis..

[9] UG4001-04-CleanPlex-SARS-CoV-2-Panel-User-guide.pdf. Accessed: Jun. 17, 2021. [Online]. Available: https://www.paragongenomics.com/wp-content/uploads/2021/02/UG4001-04-CleanPlex-SARS-CoV-2-Panel-User-guide.pdf

[10] Mwakibete, H., et al., ARTIC-NEB: SARS-CoV-2 Library PrepV.4. protocols.io, Feb. 2021. [Online]. Available: https://www.protocols.io/view/artic-neb-sars-cov-2-library-prep-br77m9rn.pdf#page=1&zoom=auto,-23,848

[11] Li, H. Aligning sequence reads, clone sequences and assembly contigs with BWA-MEM, *arXiv e-prints*, arXiv:1303.3997, (2013).

[12] Garrison, E., & Marth, G. Haplotype-based variant detection from short-read sequencing, *arXiv e-prints*, arXiv:1207.3907, (2012).

[13] Hadfield J (2018). Nextstrain: Real-time tracking of pathogen evolution. Bioinformatics.

[14] Release v13 nextstrain/ncov, GitHub. Accessed: Jan. 13, 2024. [Online]. Available: https://github.com/nextstrain/ncov/releases/tag/v13

[15] Khare S (2021). GISAID’s role in pandemic response. CCDCW.

[16] Phipson B, Smyth GK (2010). Permutation P-values should never be zero: Calculating exact P-values when permutations are randomly drawn. Stat. Appl. Genetics Mol. Biol..

[17] Pérez-Lago L (2021). Overlapping of independent SARS-CoV-2 nosocomial transmissions in a complex outbreak. mSphere.

[18] Baumgarte S (2022). Investigation of a limited but explosive COVID-19 outbreak in a German secondary school. Viruses.

[19] Haanappel CP (2023). Combining epidemiological data and whole genome sequencing to understand SARS-CoV-2 transmission dynamics in a large tertiary care hospital during the first COVID-19 wave in The Netherlands focusing on healthcare workers. Antimicrob. Resist. Infect. Control.

[20] Tonkin-Hill G (2021). Patterns of within-host genetic diversity in SARS-CoV-2. eLife.

[21] Pérez-Lago L (2021). SARS-CoV-2 superinfection and reinfection with three different strains. Transbound. Emerg. Dis..

[22] Dezordi FZ (2022). Unusual SARS-CoV-2 intrahost diversity reveals lineage superinfection. Microb. Genom.

