# Supplementary material for: Viral genome sequencing to decipher in-hospital SARS-CoV-2 transmission events
Source: Sci Rep. 2024 Mar 8;14:5768. doi: 10.1038/s41598-024-56162-7 (PMC10923895; doi:10.1038/s41598-024-56162-7)
Supplement: Supplementary file 1 — Supplementary Information. [file 41598_2024_56162_MOESM1_ESM.docx]

# SUPPLEMENTARY MATERIAL

Viral Genome Sequencing to Decipher In-Hospital SARS-CoV-2 Transmission Events

Elisabeth Esser^a,b,§^, Eva C. Schulte^a,c,d,e,f,§^, Alexander Graf^g^, Alexander Karollus^b^, Nicholas H. Smith^b^, Thomas Michler^a,h^, Stefan Dvoretskii^b^, Angel Angelov^i^, Michael Sonnabend^i^, Silke Peter^i^, Christina Engesser^i^, Aleksandar Radonic^j^, Andrea Thürmer^j^, Max von Kleist^k,l^, Friedemann Gebhardt^m^, Clarissa Prazeres da Costa^m,n^, Dirk H. Busch^m,n^, Maximilian Muenchhoff^o,n^, Helmut Blum^g^, Oliver T. Keppler^o,n^, Julien Gagneur^b,p,q,$*,^ Ulrike Protzer^a,n$*^

^a^ Institute of Virology, School of Medicine & Health, Technical University of Munich/Helmholtz Munich, Munich, Germany

^b^ School of Computation, Information and Technology, Technical University of Munich, Garching, Germany

^c^ Department of Psychiatry, University Hospital, LMU Munich, Munich, Germany

^d^ Institute of Psychiatric Phenomics and Genomics, University Hospital, LMU Munich, Munich, Germany

^e^ Department of Psychiatry, University Hospital, Medical Faculty, University of Bonn, Bonn, Germany

^f^ Institute of Human Genetics, University Hospital, Medical Faculty, University of Bonn, Bonn, Germany

^g^ Laboratory for Functional Genome Analysis, Gene Center, LMU Munich, Munich, Germany

^h^ Current address: Institute of Laboratory Medicine, University Hospital, LMU Munich, Germany

^i^ NGS Competence Center, University of Tübingen, Tübingen, Germany

^j^ Method development, research infrastructure & IT (MFI), Robert-Koch Institute (RKI), Berlin, Germany

^k^ Department of Mathematics and Computer Science, Freie Universität (FU) Berlin, Berlin, Germany

^l^ Project groups, Robert-Koch Institute, (RKI), Berlin, Germany

^m^ Institute for Medical Microbiology, Immunology and Hygiene, School of Medicine, Technical University of Munich, Munich, Germany

^n^ German Center for Infection research (DZIF), Munich partner site, Munich, Germany

^o^ Max von Pettenkofer Institute and Gene Center, Virology, National Reference Center for Retroviruses, Faculty of Medicine, LMU Munich, Munich, Germany

^p^ Institute of Human Genetics, School of Medicine, Technical University of Munich, Munich, Germany

^q^ Computational Health Center, Helmholtz Center Munich, Neuherberg, Germany

^§^ These authors contributed equally to the study and share first authorship

^$^ These authors contributed equally to the study and share last authorship

*corresponding authors: Ulrike Protzer, protzer@tum.de; Tel.: +49-89-4140-6821

Julien Gagneur, [gagneur@in.tum.de](mailto:gagneur@in.tum.de); Tel.: +49-89-289-19411

# SUPPLEMENTARY METHODS

Viral whole genome sequences were obtained from residual diagnostic material (e.g. nasopharyngeal swabs, bronchoalveolar lavage fluid, material from throat flushes, sputum, stool, or tracheal secretions) testing positive for SARS-CoV-2 by PCR. Samples were collected at Klinikum rechts der Isar in Munich, Germany, across 12 months from February 3, 2020, to January 10, 2021 (“TUM samples”, Suppl Fig 1).

An additional dataset of local viral sequences from 16 hospitals and emergency departments across Munich other than the Klinikum rechts der Isar, collected by the Ludwig Maximilian University of Munich (LMU), served as “negative” (i.e. community-based) controls. This dataset consisted of 1,156 samples collected from 1,030 individuals across a 11-month timespan from March 5, 2020, to January 10, 2021 (“LMU samples”).

Sequencing was performed on *NextSeq*, *ISeq*, or *HiSeq* platforms (Illumina, San Diego) for a total of 926 samples from 622 probands, including both patients (n=433; m=271, f=158, missing=4; age 2-99 yrs (mean ± SD: 63.8 ± 18.7 yrs)) and staff members (n=189; m=83, f=106; age 20-67 yrs (mean ± SD: 38.7 ± 12.3 yrs))
First wave samples were sequenced at the Robert Koch Institute (RKI) in Berlin, Germany. The RKI received samples covering the time period from February 3, 2020, to June 25, 2020 (n=390). For the samples sequenced at RKI, libraries were prepared using the *CleanPlex*® protocol by Paragon Genomics [1]. Since only samples with CT-values < 33.0 yielded reliable sequencing results, samples sequenced at RKI were excluded from the analysis if they had CT-values $\geq$ 33.0.
Second wave samples were sequenced at the NGS Competence Center Tübingen, Tübingen, Germany, and the Gene Center at the Ludwig Maximilian University of Munich (LMU), Munich, Germany. The NGS Competence Center Tübingen mainly sequenced samples collected between September 13, 2020, and January 10, 2021 (n=182) and the Gene Center mainly sequenced samples received for diagnostics from November 2, 2020 to January 10, 2021 (n=354). For the samples sequenced at the Gene Center of the LMU Munich and the samples sequenced at the NGS Competence Center Tübingen, the *Artic* library preparation protocol was used [2].

Sequencing reads were aligned to the SARS-CoV-2 reference genome (NC 045512.2) using *bwa-mem* [3], non-aligning reads such as those of human or bacterial origin were discarded. Primer sequences were trimmed from the mapped reads. For the consensus sequence generation, variants were called with *freebayes*. Variants were filtered using *bcftools* [4] according to a minimum mean mapping quality of the alternative allele (MQM) < 40 and a quality score (QUAL) < 10. In addition, INDELS were normalized with the *norm* command from *bcftools*. A variant was incorporated into the consensus sequence if it was supported by more than 20 reads and had at least 10 reads supporting the mutation. Positions that had a read depth below 20 reads were masked.

For the Jaccard-based clustering and longitudinal analyses, the genetic variants were called with *freebayes*, with ploidy set to 1 [5], to be able to assess all genetic variants, including variants at minor frequencies and depth. Additionally, the following quality control filters were implemented: A sample needed to reach a genome coverage over 80%, read depth at a variant position had to be higher than ten, and the number of alternate observations on the forward and the reverse strand each had to be greater than two. Applying these quality filters, 619 samples from 475 probands, containing both patients (n=320; m=207, f=112, missing=1; age 17-99 yrs (mean ± SD: 66.2 ± 17.1 yrs)) and staff members (n=155; m=66, f=89; age 20-67 yrs (mean ± SD: 38.2 ± 12.0 yrs)), were included in the final analysis.

The same quality filters were implemented for the additional dataset of local sequences, collected by the LMU, which served as negative controls. This resulted in the inclusion of 957 samples from 854 individuals (m= 285, f= 263, missing=306; age 0-102 yrs (mean ± SD: 64.8 ± 20.7 yrs), missing=18) in the analysis.

Clustering analysis was performed in *RStudio* (version 4.1.1) across the entire set of TUM samples to identify clusters of genetically related samples. Variants were filtered using *bcftools* [4] according to a minimum mean mapping quality of the alternative allele (MQM) < 40 and a quality score (QUAL) < 10. In addition, INDELS were normalized with the *norm* command from *bcftools*. Hierarchical clustering was performed using as dissimilarity the pairwise Jaccard distances computed on shared covered bases and the agglomeration method “*complete*”. The resulting multiple levels of clusters were then visualised as phylogenetic trees, which were cut, to put similar samples into groups (Suppl Fig 22). The cutting height was determined by probands, which changed their virus strain over time. The samples of these probands were assigned different *Nextstrain* clades [6] as the virus strain changed. The right cutting height was defined as the height where those samples, from the same probands with different *Nextstrain* clades, were also put into different groups by cutting the tree. The resulting groups were filtered for high similarity (Jaccard distance between samples <0.2), time of sample collection (< 30 days apart), number of probands included (≥ three probands). The Jaccard distance was chosen as a distance measure as it is a measure of similarity of whole datasets and thus, well-suited to compute the similarity of two samples (each sample with the according genetic variants as one dataset). After investigation of various cutoffs, we set a Jaccard distance cutoff to <0.2 as it gave reasonable clusters. With a cutoff value of <0.1, for example, we observed that a sample of the same patient would not be included in the cluster with the other samples from this patient. When relaxing the cutoff to <0.3, samples that differed from the rest of the cluster by five genetic variants were included. It is unlikely that a sample with this genetic divergence is part of the same infection chain. The 30-day time window was chosen based on a conservative estimate that any two positive samples more than 30 days apart would unlikely stem from the same chain of infections. Based on the viral strains circulating at the time, it was assumed that–in the vast majority of cases–an infectious period would be <=15 days and an incubation period for the next infected individual would also be <=15 days [7], [8], [9]. This was implemented by computing a mean date for each cluster and excluding all samples that deviated more than 30 days from this mean date. Genetic variants were only included in the analysis if they occurred in at least one sample with an allele frequency higher than 50%. In the interest of detailed visualization, two plots were created for each cluster (Suppl Fig 7 to 20). The plots in part B of the figures include low-frequency variants, which show that in many clusters some samples do not share the consensus genetic variant, but still share the genetic variant at lower frequencies. These low-frequency variants were never used for any computations in the clustering analysis.

As part of routine procedures, interview-based contact tracing was performed by a physician specialising in clinical hygiene whenever a transmission cluster was suspected clinically. Individuals were included in an interview-based transmission cluster when either a close contact was reported, defined as exposure to presumably high concentrations of aerosols without wearing an FFP2 mask or if the index or the contact were wearing no more than a surgical mask while conversing or being in close proximity for more than ten minutes (Fig 1B), or when a patient had shared a room with the index patient at some point, regardless of the duration or the use of personal protective equipment (Fig. 1B). In addition to either of the inclusion criteria above, the infections needed to have taken place within the potentially infectious period of the index patient (defined as two days before until 10 days after symptom onset; for asymptomatic cases, up to 10 days after the date of the first positive test). In cases of uncertain routes of infection (community-acquired vs. while working as a health care provider), professional exposure was assumed, and the individuals were always included in the clusters. A cluster was defined when at least three infections were connected as described above.

To validate the in-hospital transmissions, September 13, 2020, to January 10, 2021, was chosen as a time period with enough comparable data for both the TUM and the LMU datasets. For the last viral sequence of each individual in the TUM dataset (n=334) during this period, the number k_i_ of individuals with a closely related virus genotype (Jaccard distance <0.2) as well as the average (T_k_) for the entire TUM dataset was computed. To compare our data to an out-of-hospital “negative” control, we proceeded with Monte Carlo simulations by randomly drawing n=334 viral genomes from the LMU dataset (n=681, only last samples from each individual), collected at other local hospitals and emergency departments during the same time period, repeating this process 1,000 times and every time computing the number k_i_^LMU^ of closely related individuals for each sample of the TUM dataset. As before, the average T_k_^LMU^ was computed each time.

A similar approach was used to confirm true transmission clusters ruling out multiple simultaneous introductions. For each Jaccard-based cluster, the number of closely related samples from a random draw (n=343) of the LMU dataset (n=681) was computed and the process was repeated 1,000 times for each cluster. If this number was smaller than the number of samples in the Jaccard-based cluster in 95% of the results, the Jaccard-based cluster was deemed likely to be an in-hospital transmission cluster. The higher number of samples in the random draw compared to the previous simulation results from the fact that if multiple samples of a single individual were assigned to different Jaccard-based clusters, all of these samples were included in the analysis.

# SUPPLEMENTARY FIGURES


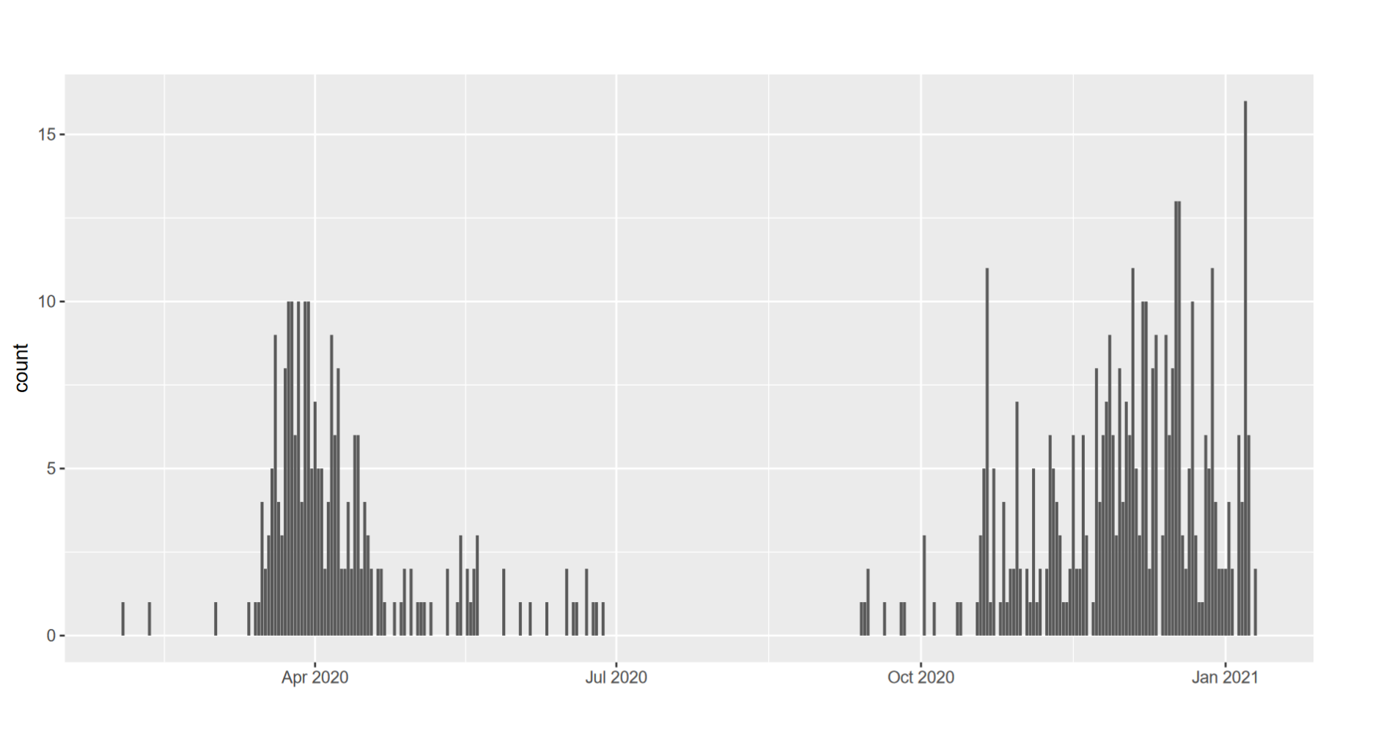
Suppl. Fig.1: Distribution of samples over one year. Number of samples collected at Klinikum rechts der Isar in Munich, Germany, per day across a 12-month timespan from February 3, 2020, to January 10, 2021, which met the quality criteria and were included in the analysis.


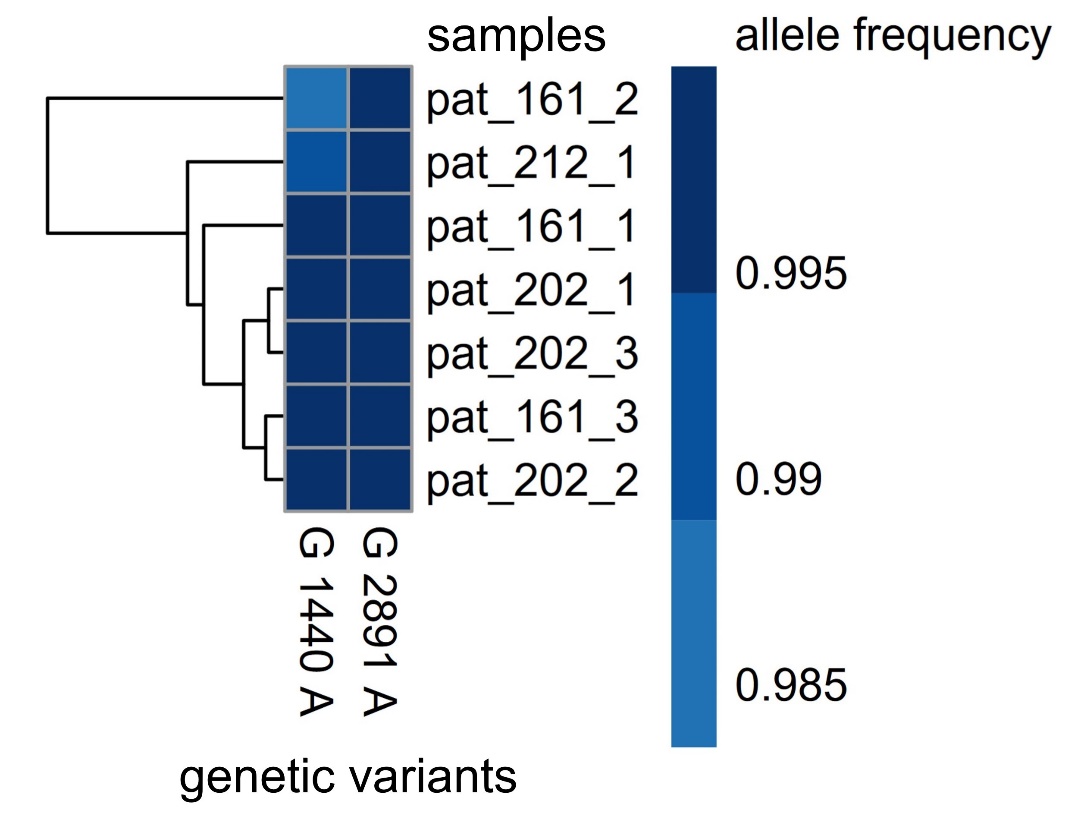

Suppl. Fig.2: Cluster A. This cluster includes seven samples from three patients. The color gradient depicts allele frequencies, grey coloring denotes that the base call did not pass the quality control filters for a given variant. X-axes display genetic variants with allele frequencies >50% in at least one sample when compared to the reference genome (NC 045512.2) Each row denotes one sample included in the cluster.


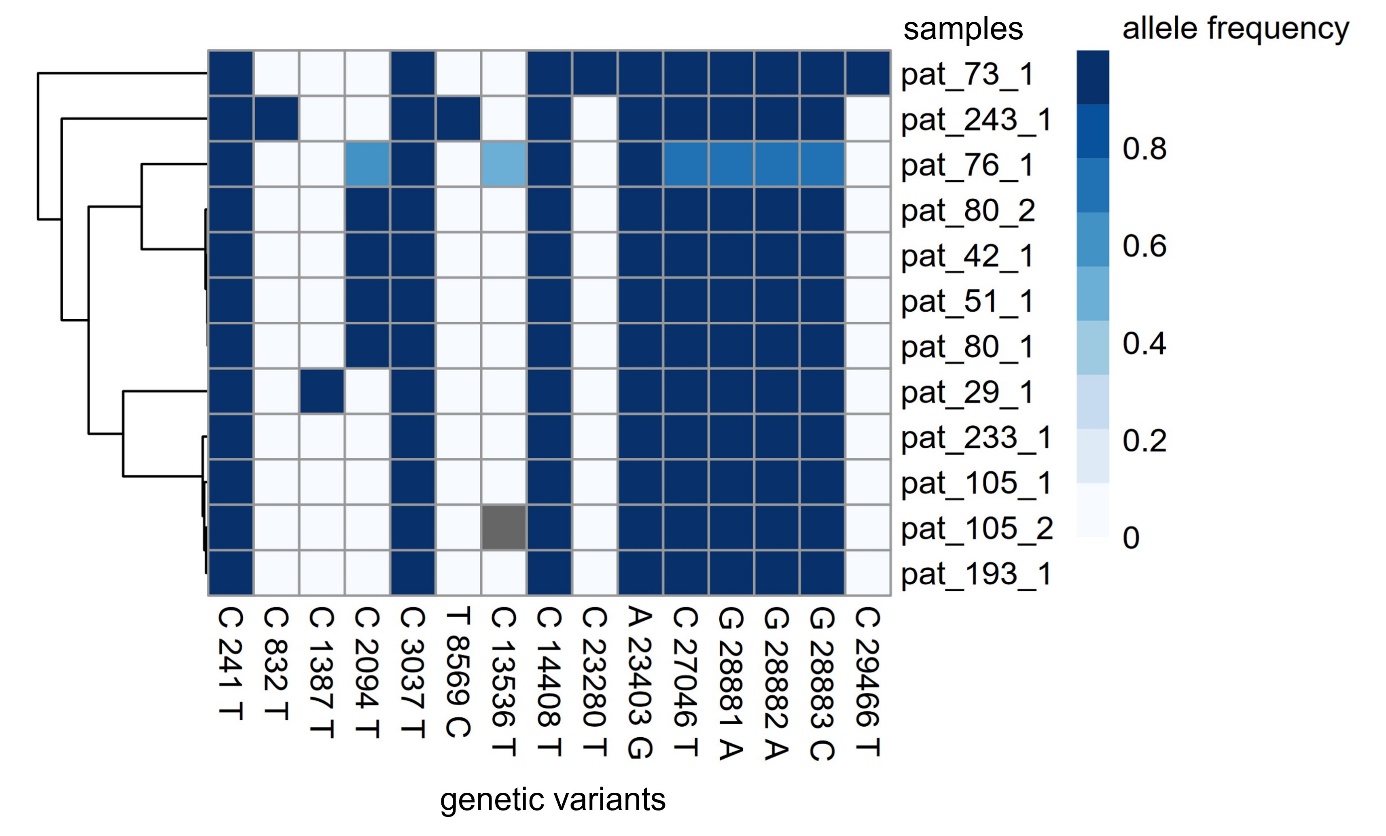

Suppl. Fig.3: Cluster B. This Cluster includes twelve samples from ten probands, five staff members and five patients. The color gradient depicts allele frequencies, grey coloring denotes that the base call did not pass the quality control filters for a given variant. X-axes display genetic variants with allele frequencies >50% in at least one sample when compared to the reference genome (NC 045512.2) Each row denotes one sample included in the cluster.


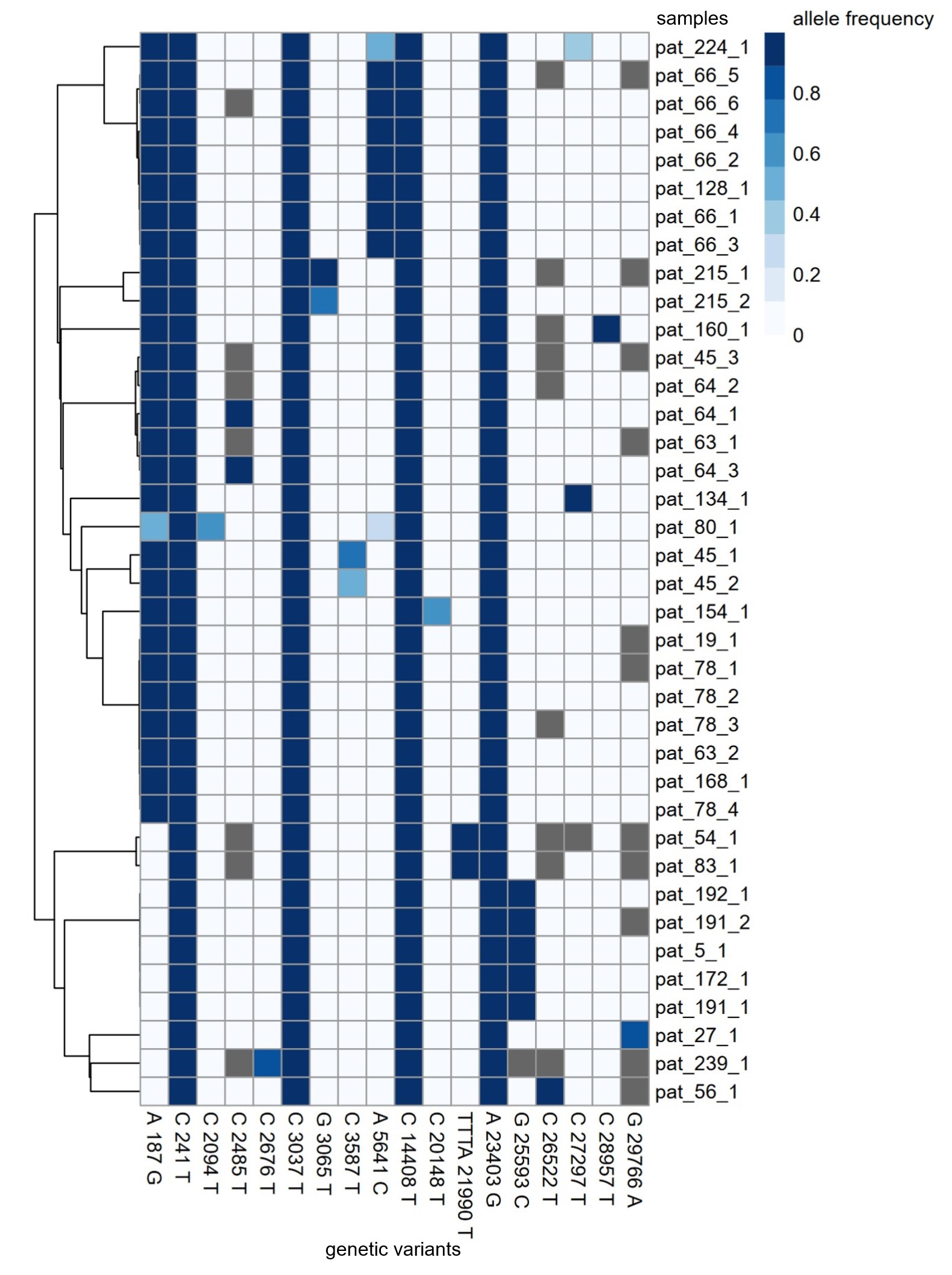

Suppl. Fig.4: Cluster C. This cluster includes 38 samples from 23 probands (two staff members and 21 patients). This cluster is bigger and more heterogenous because the defining genetic variants in this cluster (C 241 T, C 3037 T, C 14408 T, A 23403 G) are four variants common to most viruses circulating at the time. Since this makes it unlikely that all samples with those genetic variants originated from one big transmission event, there was an extra filter implemented for this cluster. The filter only includes samples in the cluster that have additional genetic variants to the four common ones. The color gradient depicts allele frequencies, grey coloring denotes that the base call did not pass the quality control filters for a given variant. X-axes display genetic variants with allele frequencies >50% in at least one sample when compared to the reference genome (NC 045512.2) Each row denotes one sample included in the cluster.


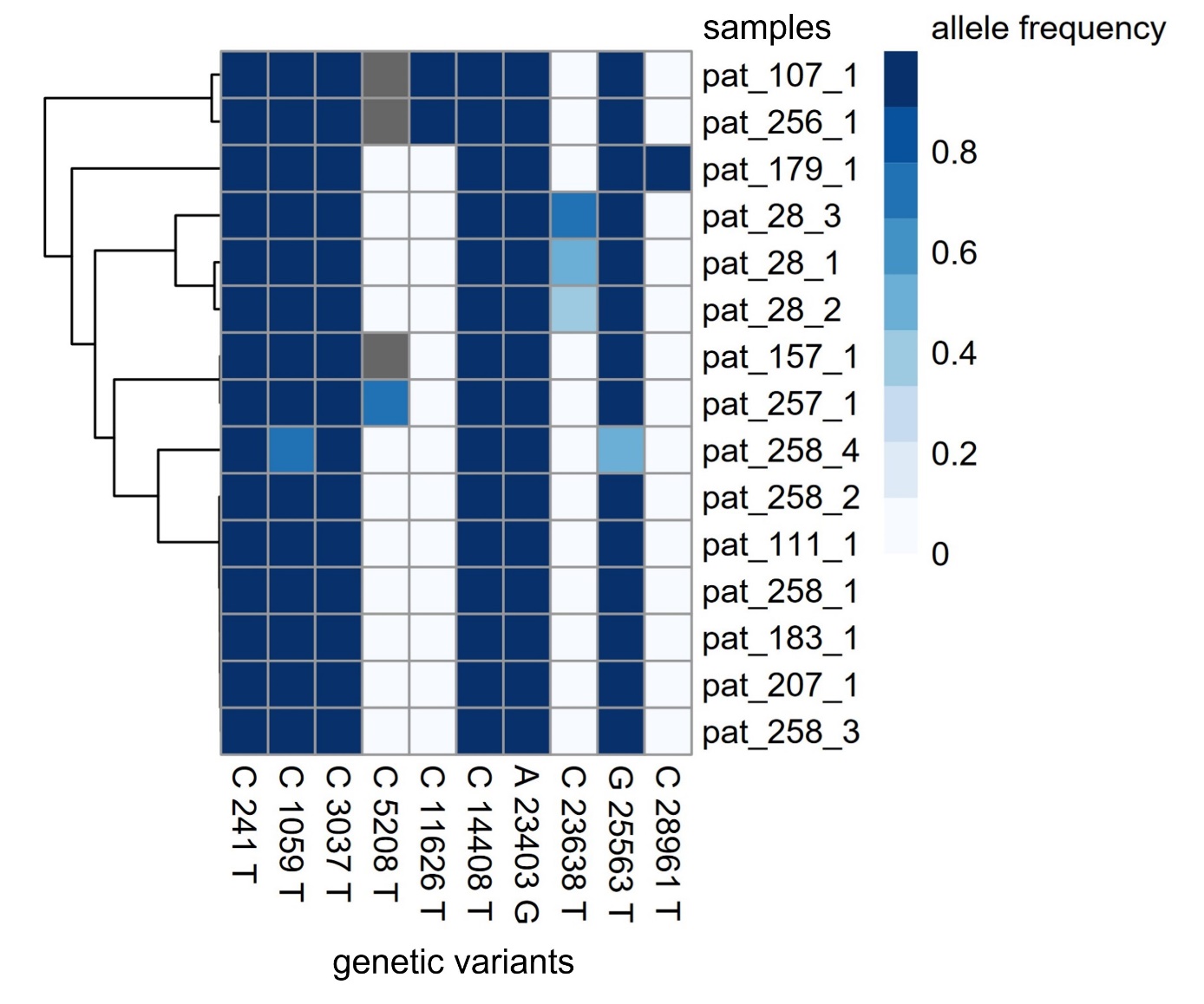

Suppl. Fig.5: Cluster D. This cluster includes 15 samples from ten probands (three staff members and seven patients). The color gradient depicts allele frequencies, grey coloring denotes that the base call did not pass the quality control filters for a given variant. X-axes display genetic variants with allele frequencies >50% in at least one sample when compared to the reference genome (NC 045512.2) Each row denotes one sample included in the cluster.


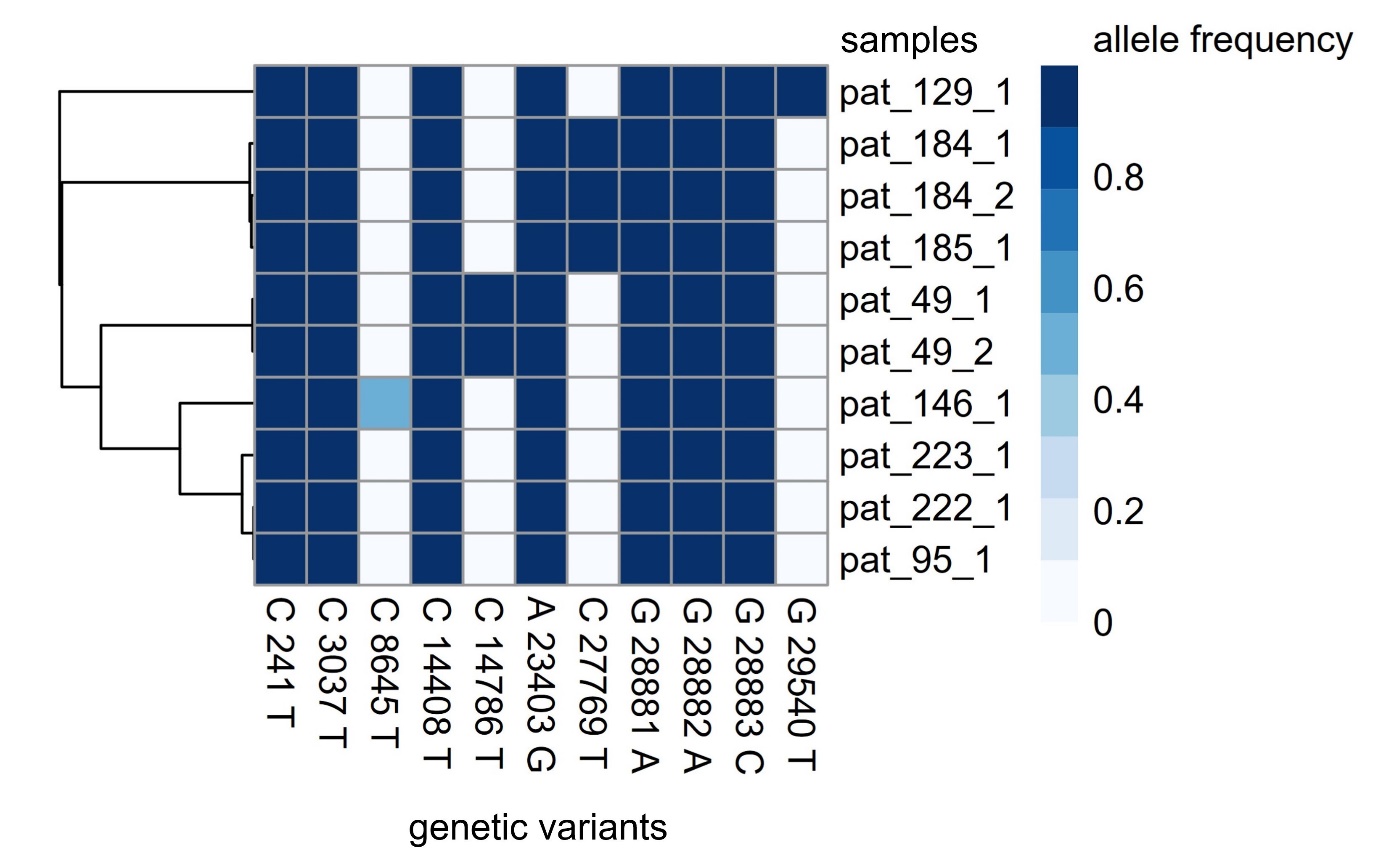

Suppl. Fig.6: Cluster E. This cluster includes ten samples from eight patients. The color gradient depicts allele frequencies, grey coloring denotes that the base call did not pass the quality control filters for a given variant. X-axes display genetic variants with allele frequencies >50% in at least one sample when compared to the reference genome (NC 045512.2) Each row denotes one sample included in the cluster.


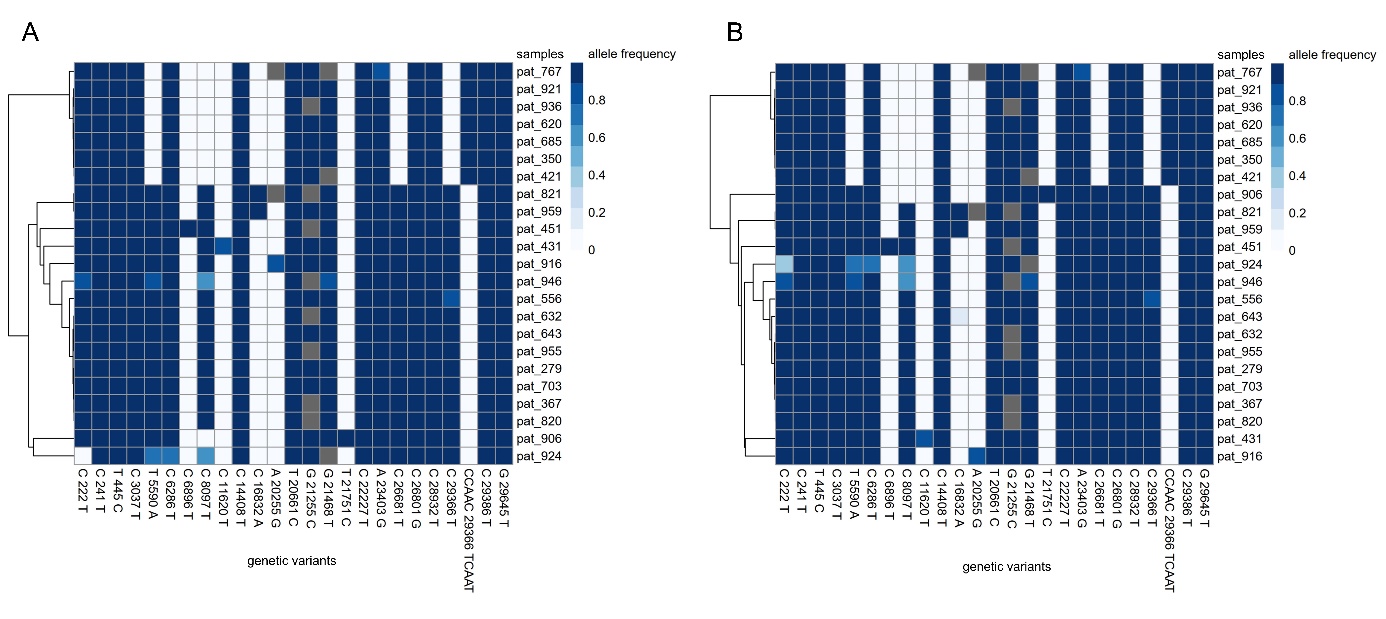

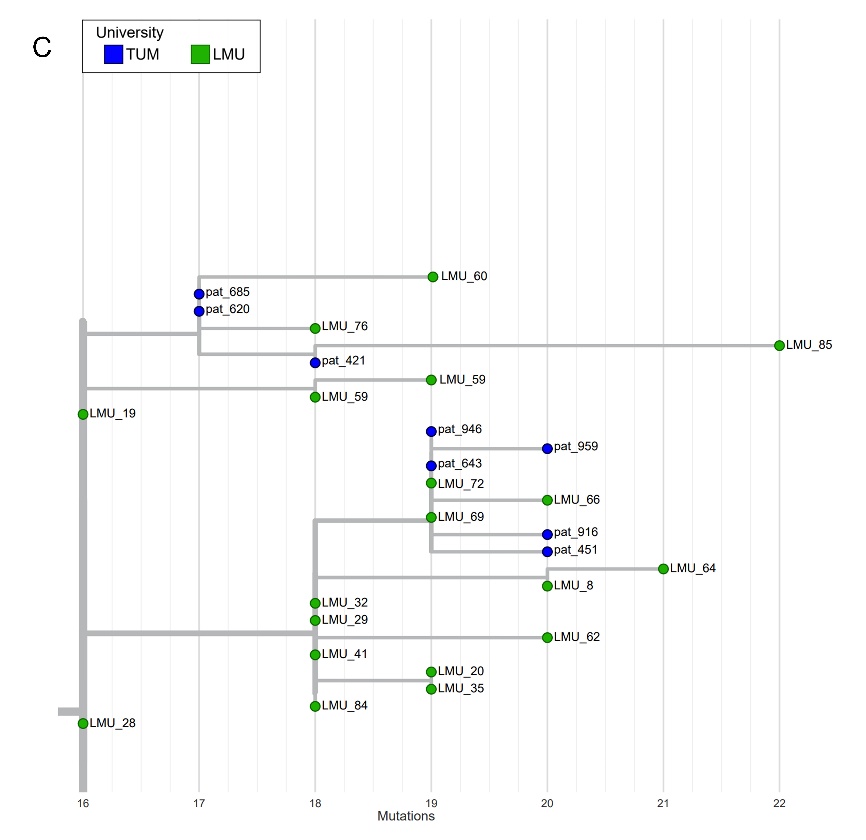


Suppl. Fig.7: Cluster F. (A) This cluster includes 23 samples from 23 probands (nine staff members and twelve patients). The color gradient depicts allele frequencies, grey coloring denotes that the base call did not pass the quality control filters for a given variant. X-axes display genetic variants with allele frequencies >50% in at least one sample when compared to the reference genome (NC 045512.2) Each row denotes one sample included in the cluster. (B) Cluster F with low-frequency variants visible. (C) Representation of Cluster F in the phylogenetic tree. Eight of the 23 samples were included in the tree.


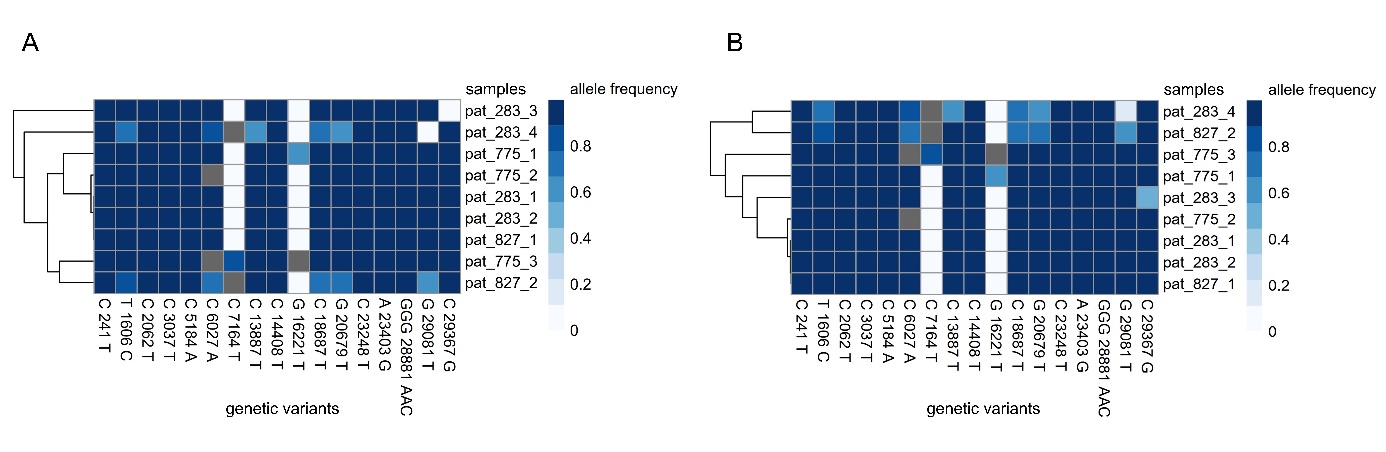

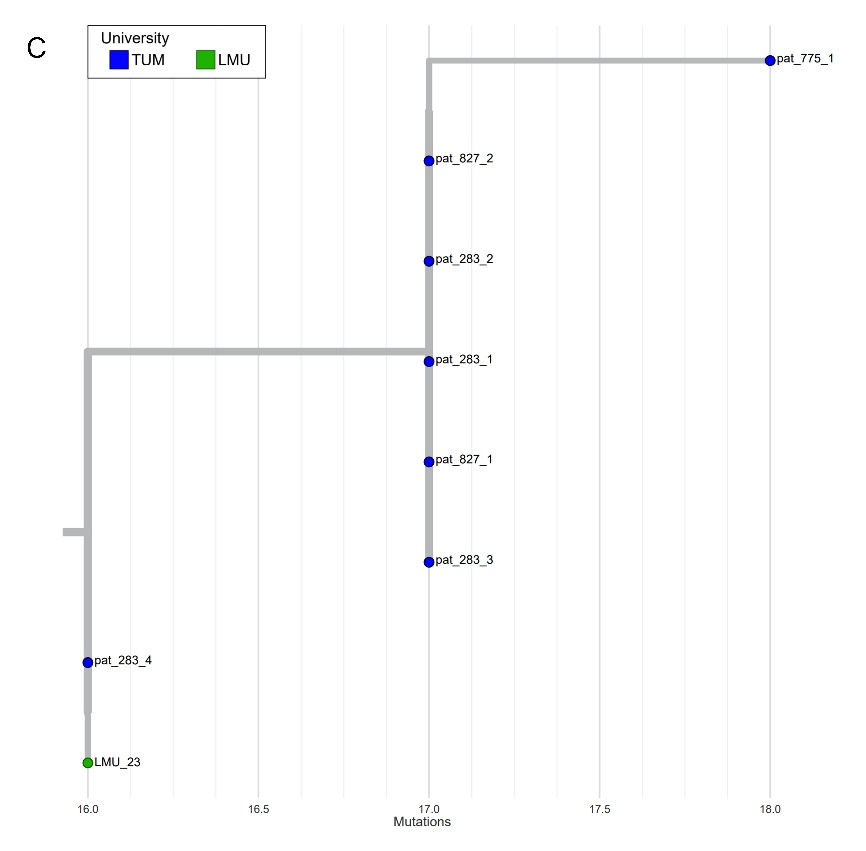

Suppl. Fig.8: Cluster G. (A) This cluster includes nine samples from three patients. The color gradient depicts allele frequencies, grey coloring denotes that the base call did not pass the quality control filters for a given variant. X-axes display genetic variants with allele frequencies >50% in at least one sample when compared to the reference genome (NC 045512.2) Each row denotes one sample included in the cluster. (B) Cluster G with low-frequency variants visible. (C) Representation of Cluster G in the phylogenetic tree. Seven of the nine samples were included in the tree.


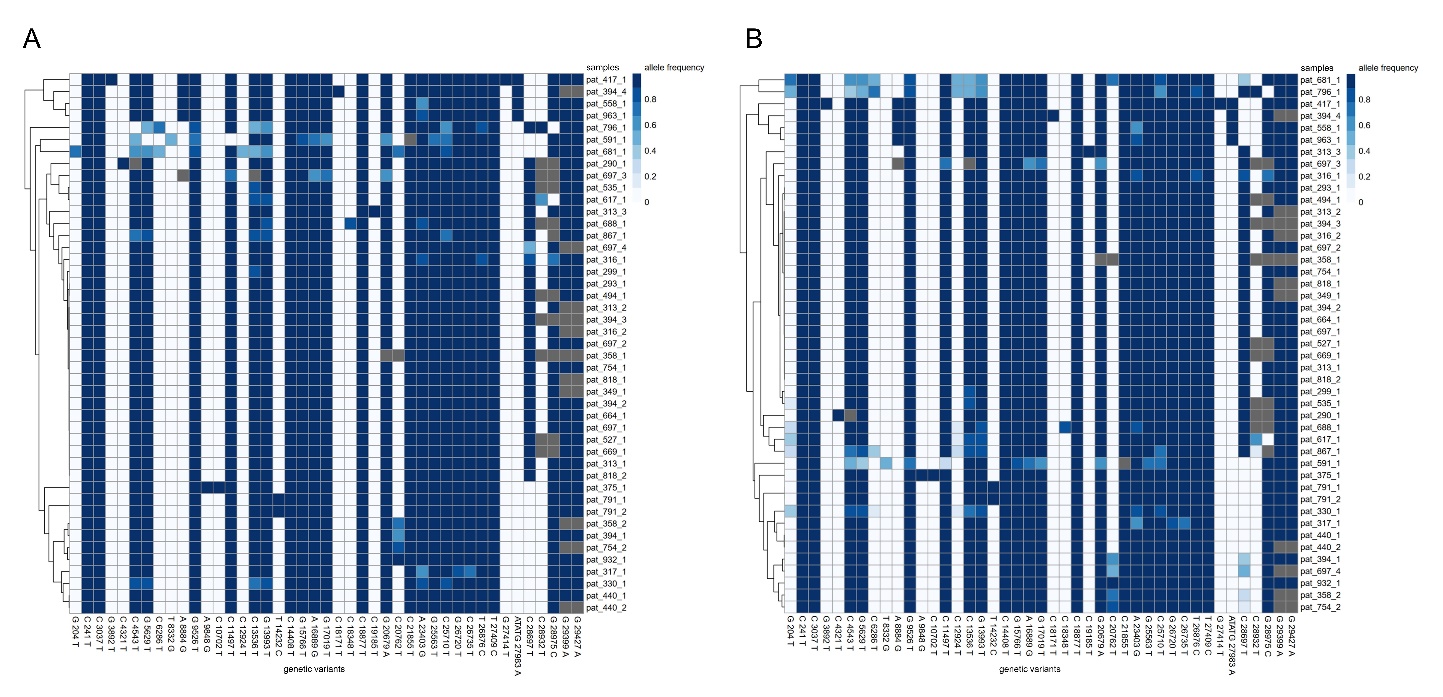

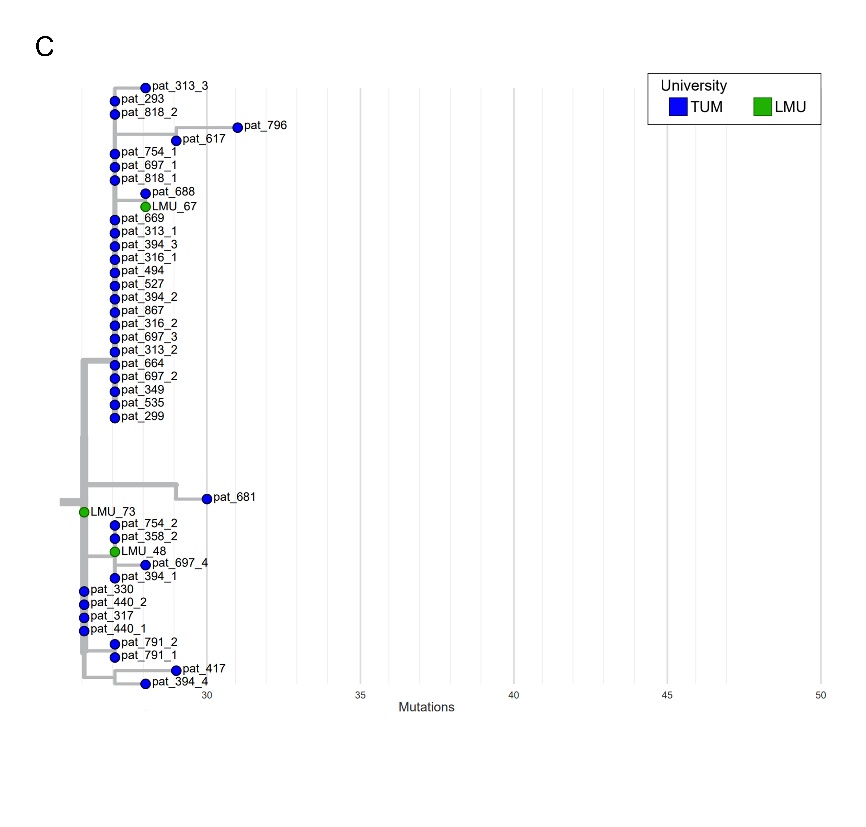


Suppl. Fig.9: Cluster H. (A) This cluster includes 45 samples from 31 probands (eleven staff members and 20 patients). The color gradient depicts allele frequencies, grey coloring denotes that the base call did not pass the quality control filters for a given variant. X-axes display genetic variants with allele frequencies >50% in at least one sample when compared to the reference genome (NC 045512.2) Each row denotes one sample included in the cluster. (B) Cluster H with low-frequency variants visible. (C) Representation of Cluster H in the phylogenetic tree. 38 of the 45 samples were included in the tree.


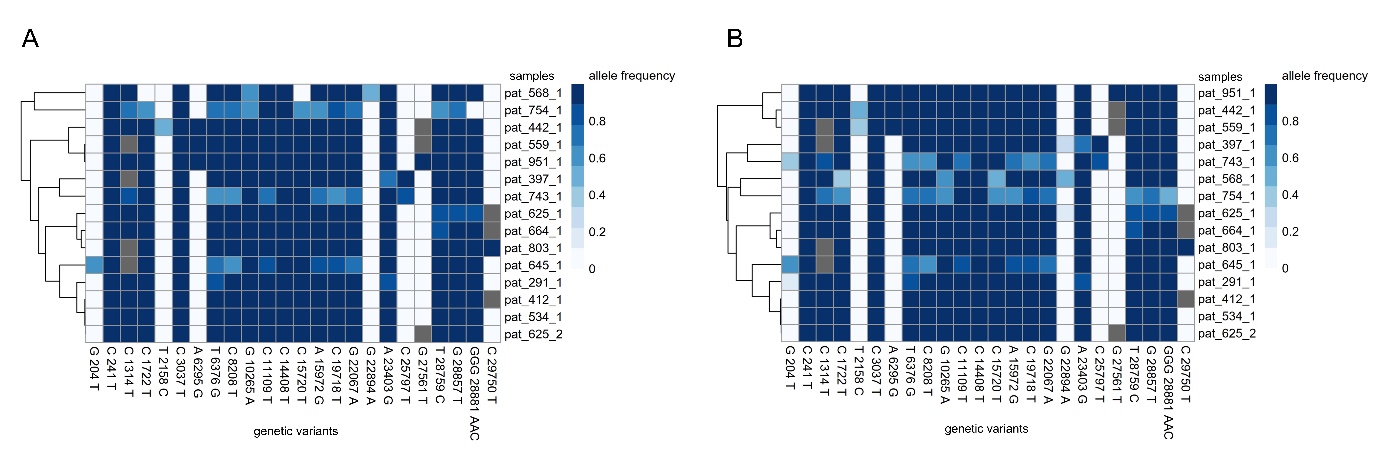

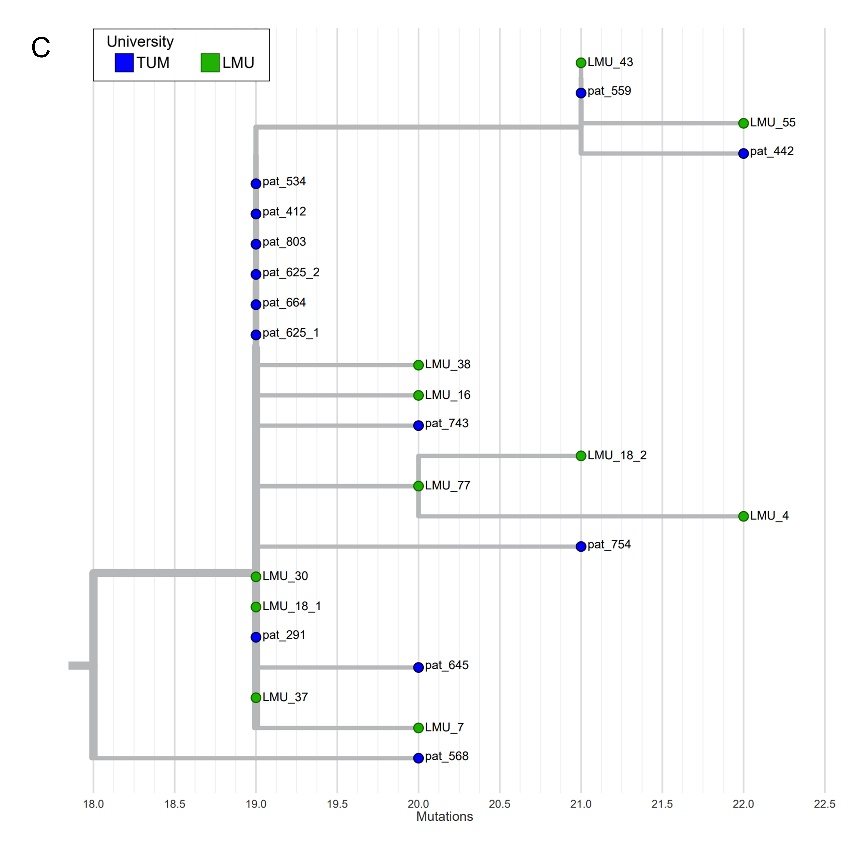

Suppl. Fig.10: Cluster I. (A) This cluster includes 15 samples from 14 probands, three staff members and eleven patients. The color gradient depicts allele frequencies, grey coloring denotes that the base call did not pass the quality control filters for a given variant. X-axes display genetic variants with allele frequencies >50% in at least one sample when compared to the reference genome (NC 045512.2) Each row denotes one sample included in the cluster. (B) Cluster I with low-frequency variants visible. (C) Representation of Cluster I in the phylogenetic tree. 13 of the 15 samples were included in the tree.


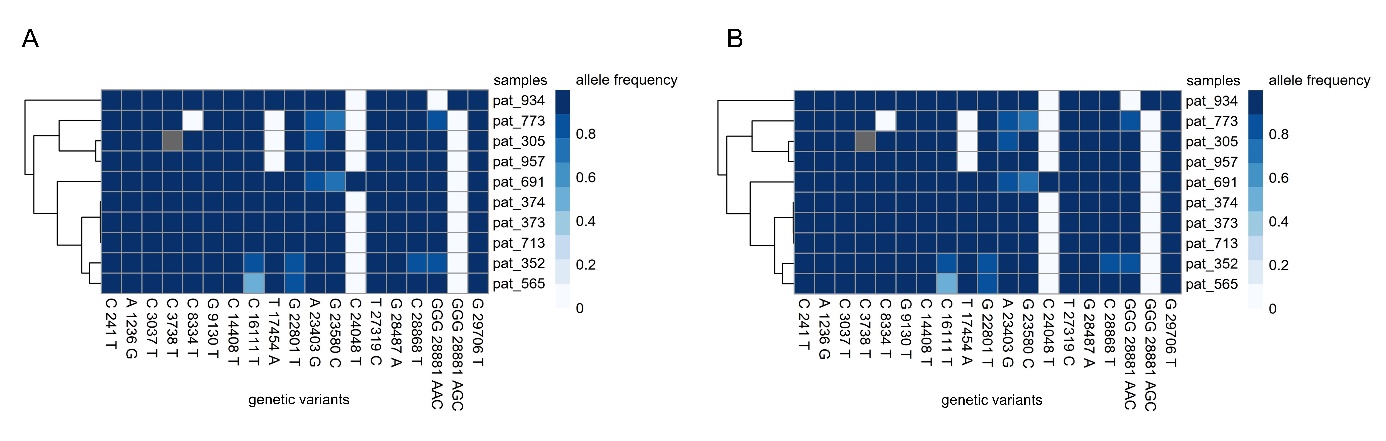

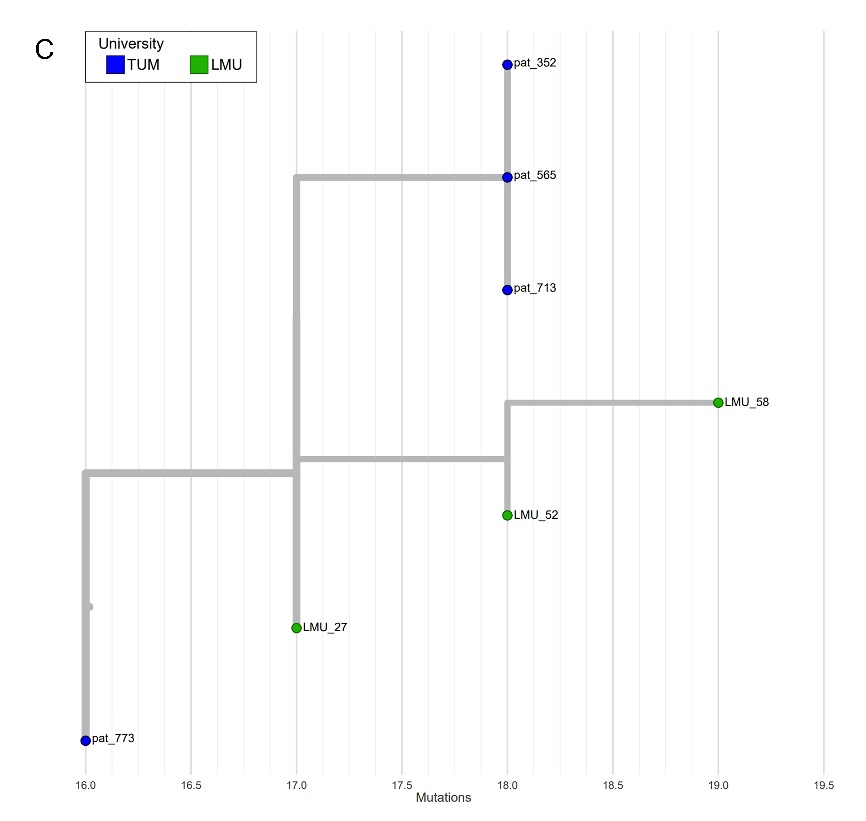

Suppl. Fig. 11: Cluster J. (A) This cluster includes ten samples from ten probands, five staff members and five patients. The color gradient depicts allele frequencies, grey coloring denotes that the base call did not pass the quality control filters for a given variant. X-axes display genetic variants with allele frequencies >50% in at least one sample when compared to the reference genome (NC 045512.2) Each row denotes one sample included in the cluster. (B) Cluster J with low-frequency variants visible. (C) Representation of Cluster J in the phylogenetic tree. Four of the ten samples were included in the tree.


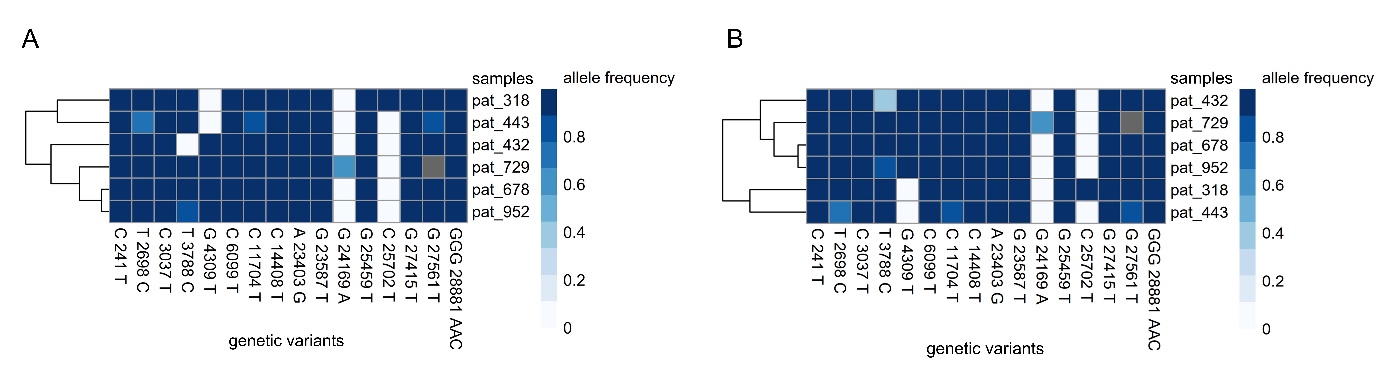

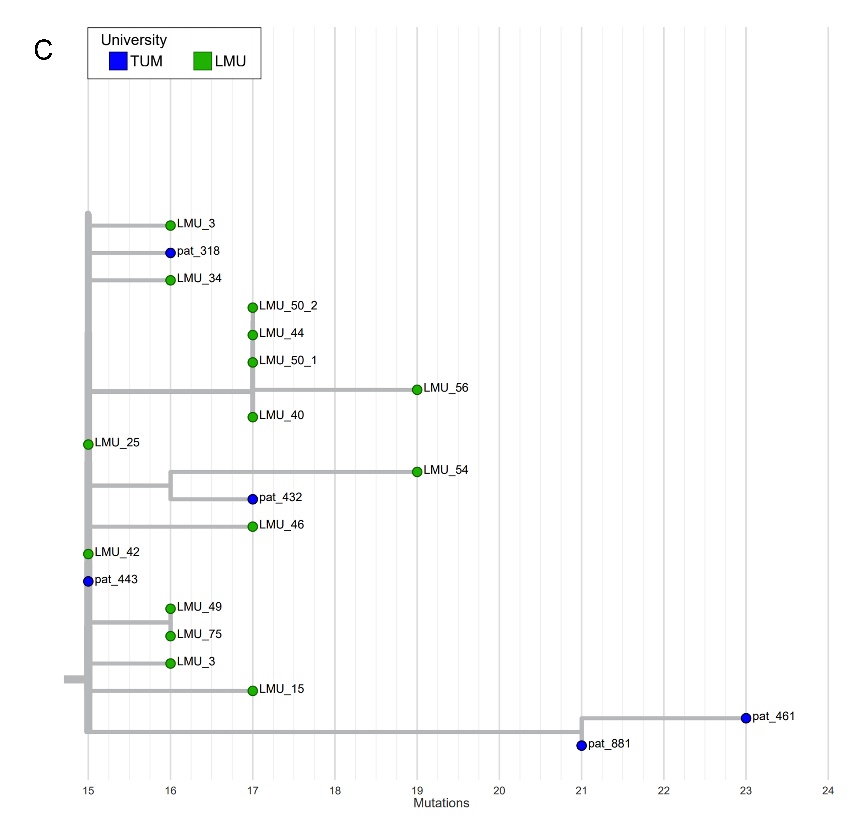

Suppl. Fig.12: Cluster K. (A) This cluster includes six samples from six probands, two staff members and four patients. The color gradient depicts allele frequencies, grey coloring denotes that the base call did not pass the quality control filters for a given variant. X-axes display genetic variants with allele frequencies >50% in at least one sample when compared to the reference genome (NC 045512.2) Each row denotes one sample included in the cluster. (B) Cluster K with low-frequency variants visible. (C) Representation of Cluster K in the phylogenetic tree. Three of the six samples were included in the tree.


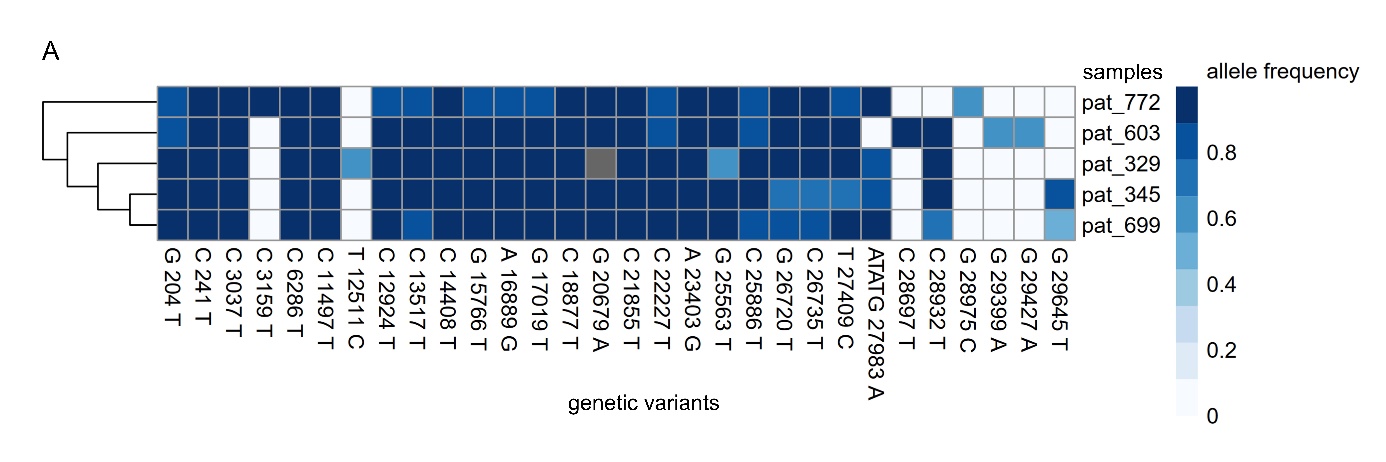

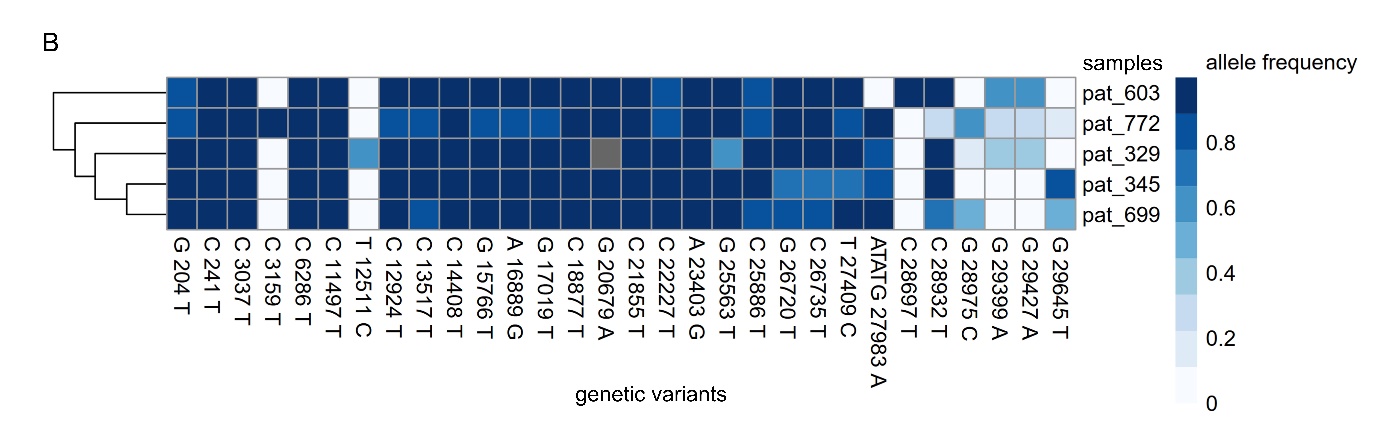

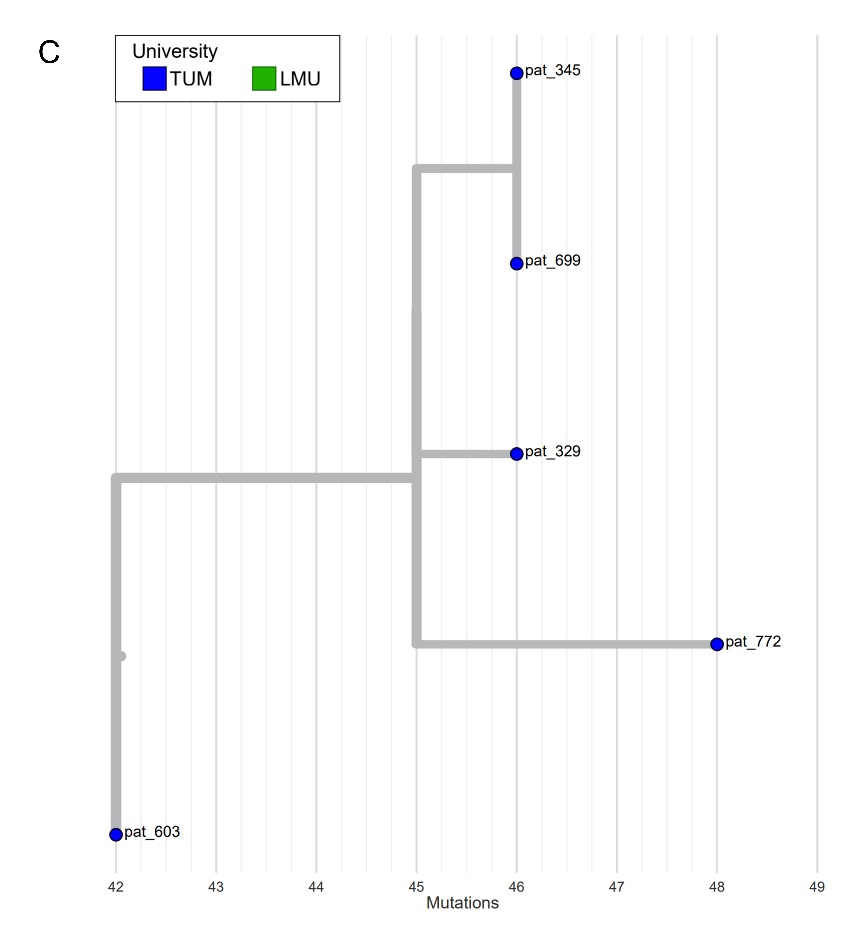

Suppl. Fig.13: Cluster L. (A) This cluster includes five samples from five probands, one staff member and four patients. The color gradient depicts allele frequencies, grey coloring denotes that the base call did not pass the quality control filters for a given variant. X-axes display genetic variants with allele frequencies >50% in at least one sample when compared to the reference genome (NC 045512.2) Each row denotes one sample included in the cluster. (B) Cluster L with low-frequency variants visible. (C) Representation of Cluster L in the phylogenetic tree. Five of the five samples were included in the tree.


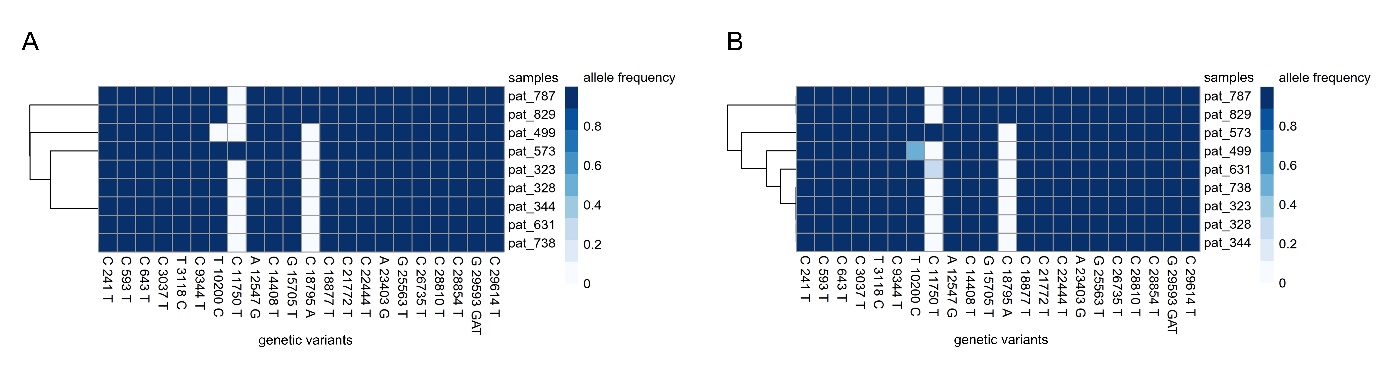

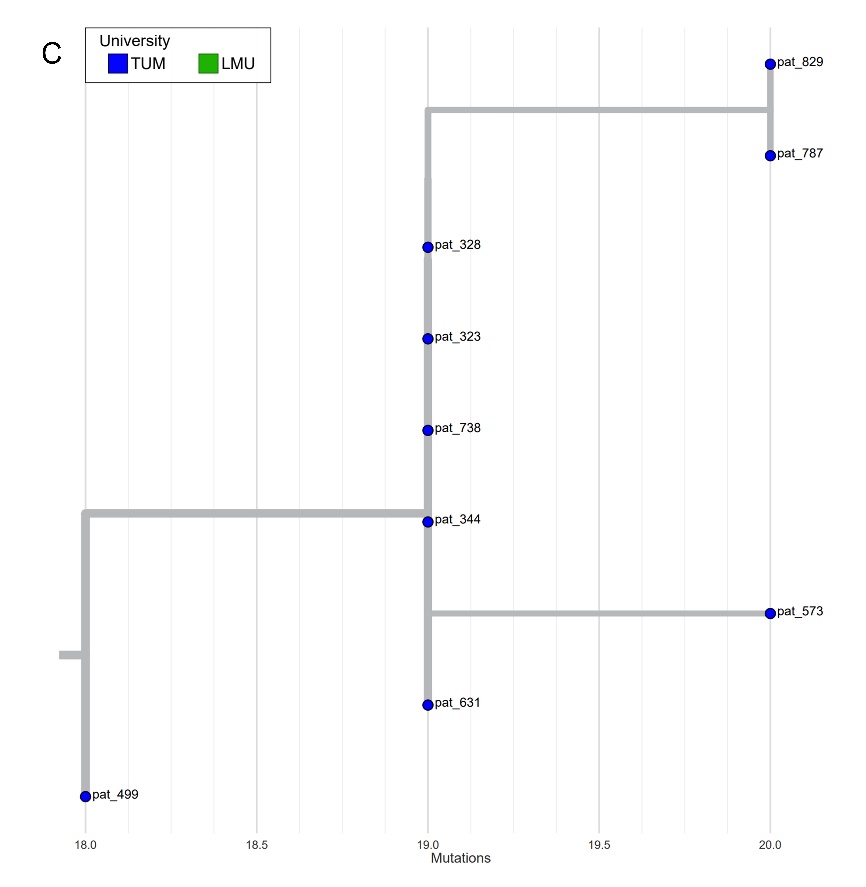


Suppl. Fig 14: Cluster M. (A) This cluster includes nine samples from nine individuals working in a non-health-care-related sector that very likely infected each. The color gradient depicts allele frequencies, grey coloring denotes that the base call did not pass the quality control filters for a given variant. X-axes display genetic variants with allele frequencies >50% in at least one sample when compared to the reference genome (NC 045512.2) Each row denotes one sample included in the cluster. (B) Cluster M with low-frequency variants visible. (C) Representation of Cluster M in the phylogenetic tree. Nine of the nine samples were included in the tree.


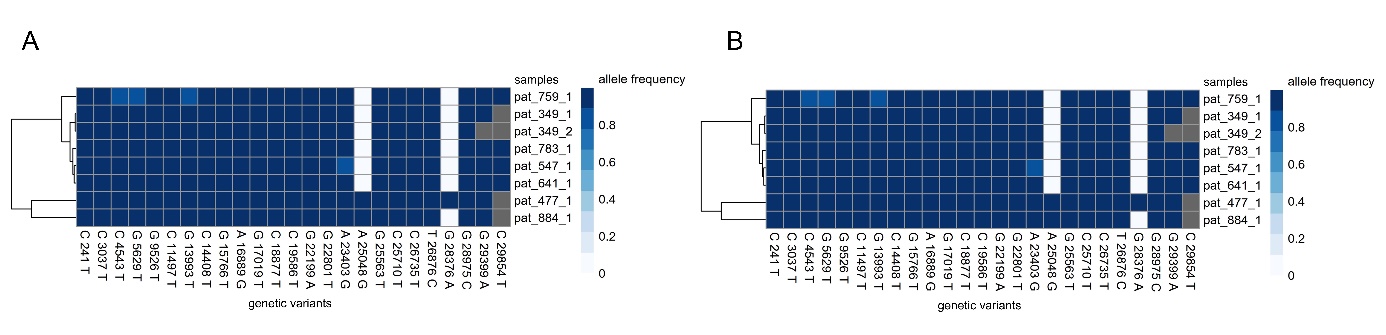

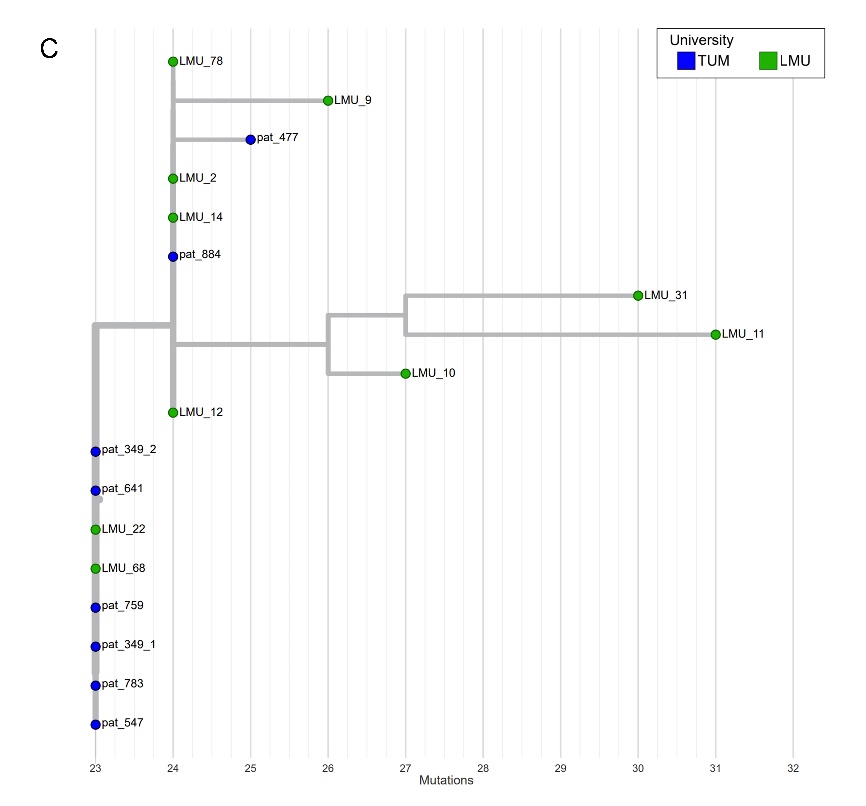

Suppl. Fig.15: Cluster N. (A) This cluster includes eight samples from seven probands, three staff members and four patients. The color gradient depicts allele frequencies, grey coloring denotes that the base call did not pass the quality control filters for a given variant. X-axes display genetic variants with allele frequencies >50% in at least one sample when compared to the reference genome (NC 045512.2) Each row denotes one sample included in the cluster. (B) Cluster N with low-frequency variants visible. (C) Representation of Cluster N in the phylogenetic tree. Eight of the eight samples were included in the tree.


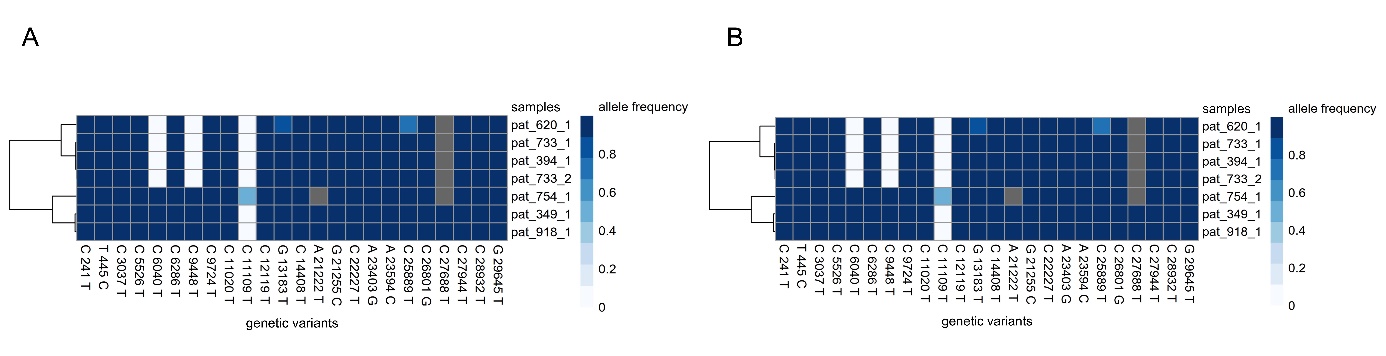

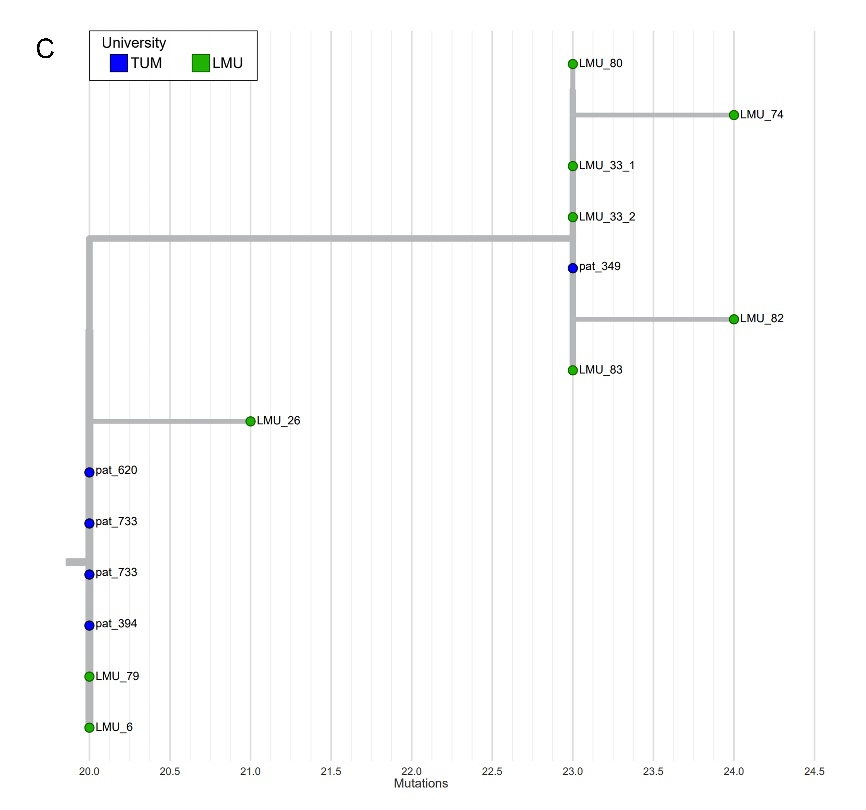

Suppl. Fig.16: Cluster O. (A) This cluster includes seven samples from six probands, one staff member and five patients. The color gradient depicts allele frequencies, grey coloring denotes that the base call did not pass the quality control filters for a given variant. X-axes display genetic variants with allele frequencies >50% in at least one sample when compared to the reference genome (NC 045512.2) Each row denotes one sample included in the cluster. (B) Cluster O with low-frequency variants visible. (C) Representation of Cluster O in the phylogenetic tree. Five of the seven samples were included in the tree.


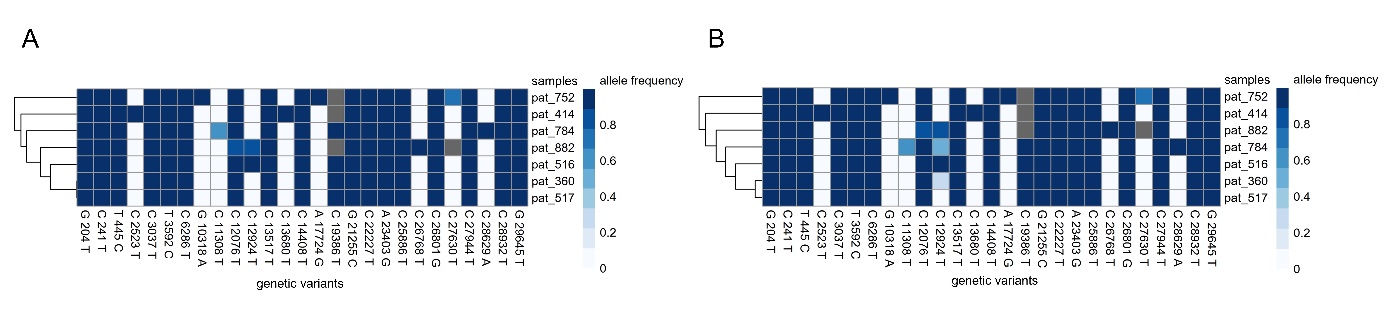

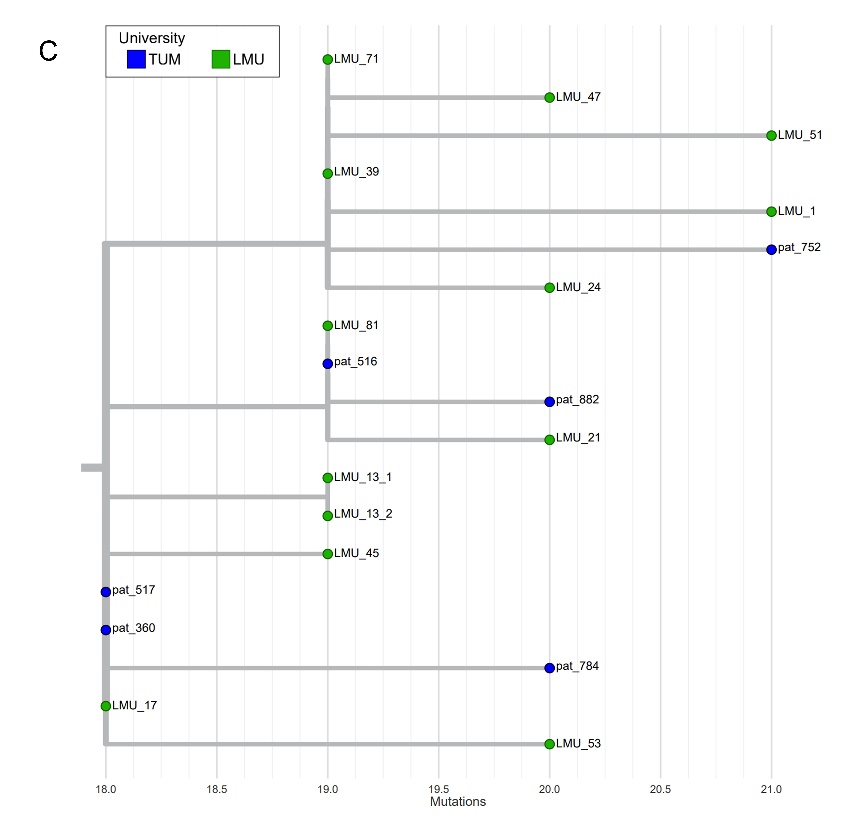

Suppl. Fig.17: Cluster P. (A) This cluster includes seven samples from seven probands, three staff members and four patients. The color gradient depicts allele frequencies, grey coloring denotes that the base call did not pass the quality control filters for a given variant. X-axes display genetic variants with allele frequencies >50% in at least one sample when compared to the reference genome (NC 045512.2) Each row denotes one sample included in the cluster. (B) Cluster P with low-frequency variants visible. (C) Representation of Cluster P in the phylogenetic tree. Six of the seven samples were included in the tree.


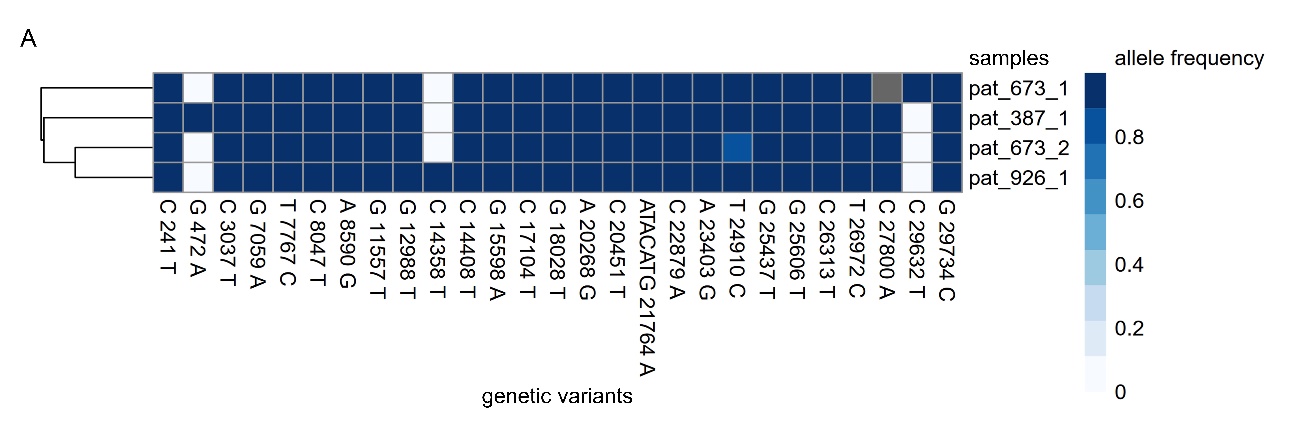

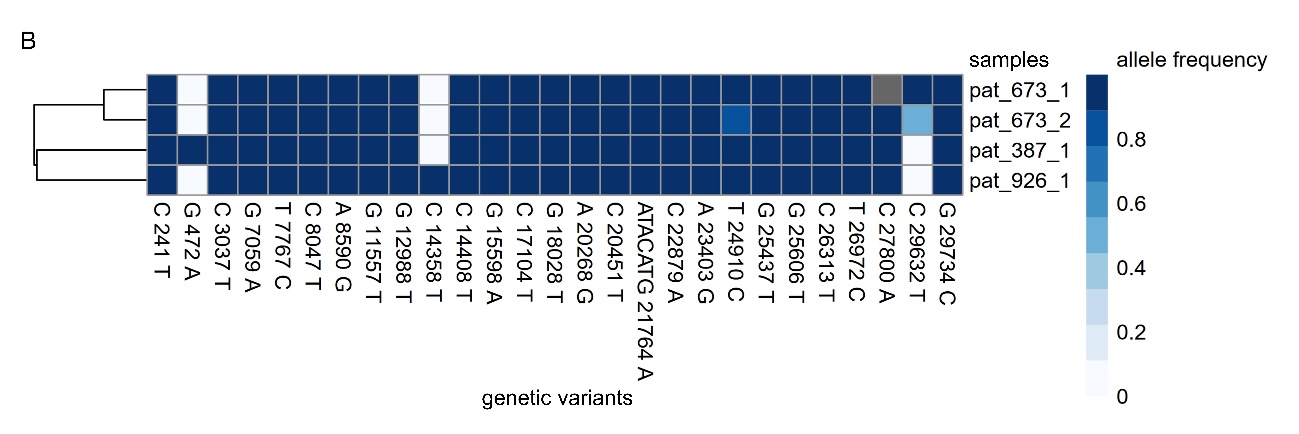

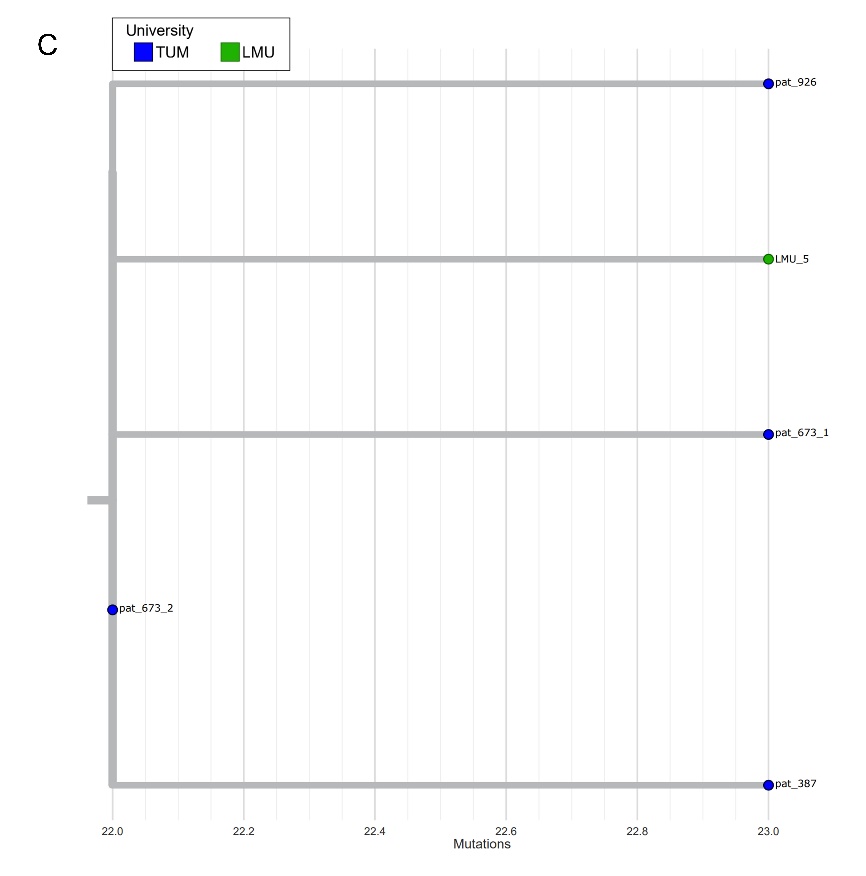

Suppl. Fig.18: Cluster Q. (A) This cluster includes four samples from three probands, one staff member and one patient. The color gradient depicts allele frequencies, grey coloring denotes that the base call did not pass the quality control filters for a given variant. X-axes display genetic variants with allele frequencies >50% in at least one sample when compared to the reference genome (NC 045512.2) Each row denotes one sample included in the cluster. (B) Cluster Q with low-frequency variants visible. (C) Representation of Cluster Q in the phylogenetic tree. Four of the four samples were included in the tree.


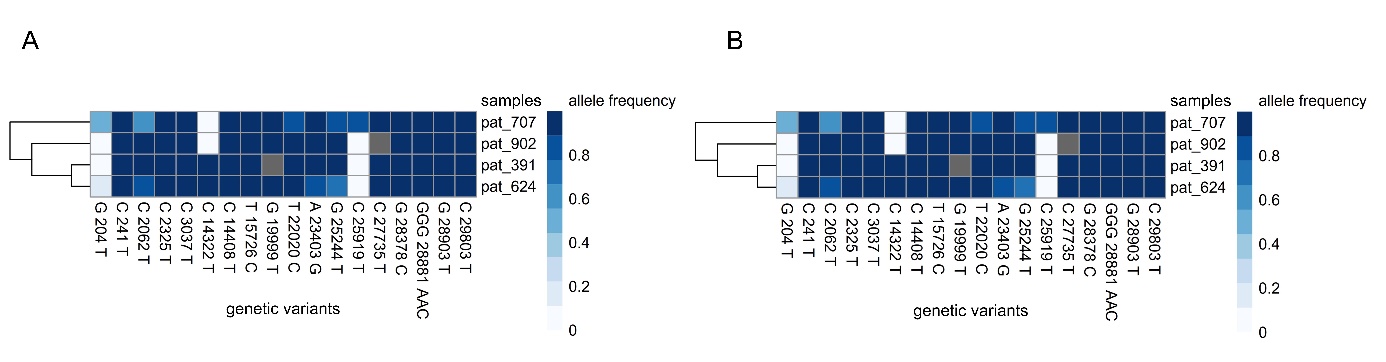

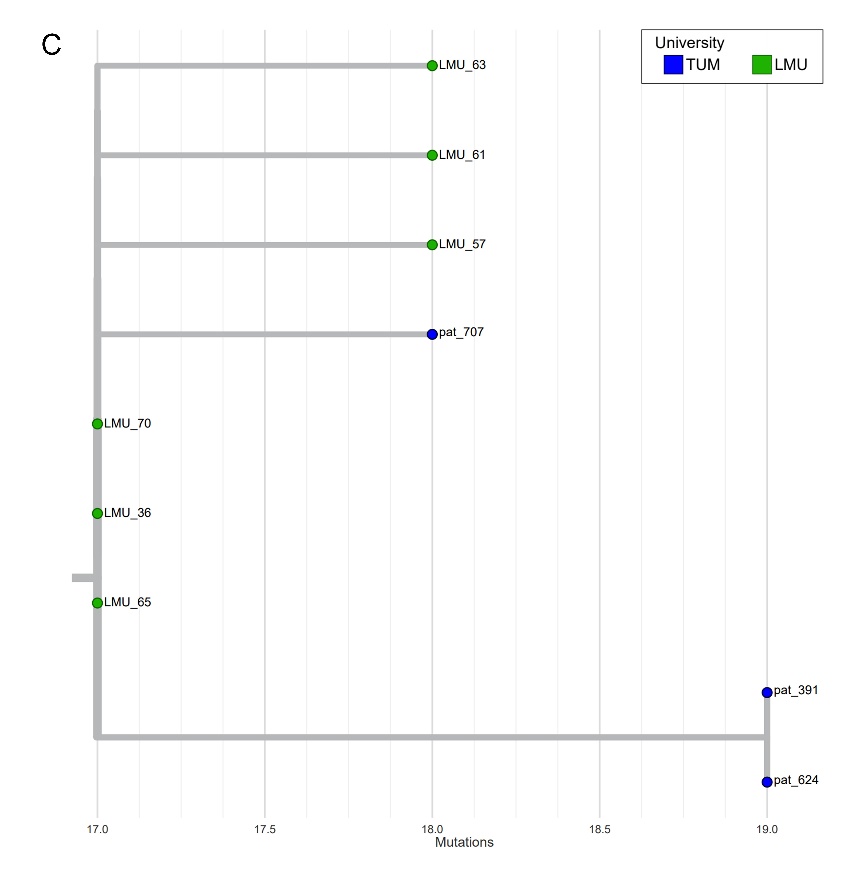

Suppl. Fig.19: Cluster R. (A) This cluster includes four samples from four probands, two staff members and two patients. The color gradient depicts allele frequencies, grey coloring denotes that the base call did not pass the quality control filters for a given variant. X-axes display genetic variants with allele frequencies >50% in at least one sample when compared to the reference genome (NC 045512.2) Each row denotes one sample included in the cluster. (B) Cluster R with low-frequency variants visible. (C) Representation of Cluster R in the phylogenetic tree. Three of the four samples were included in the tree.


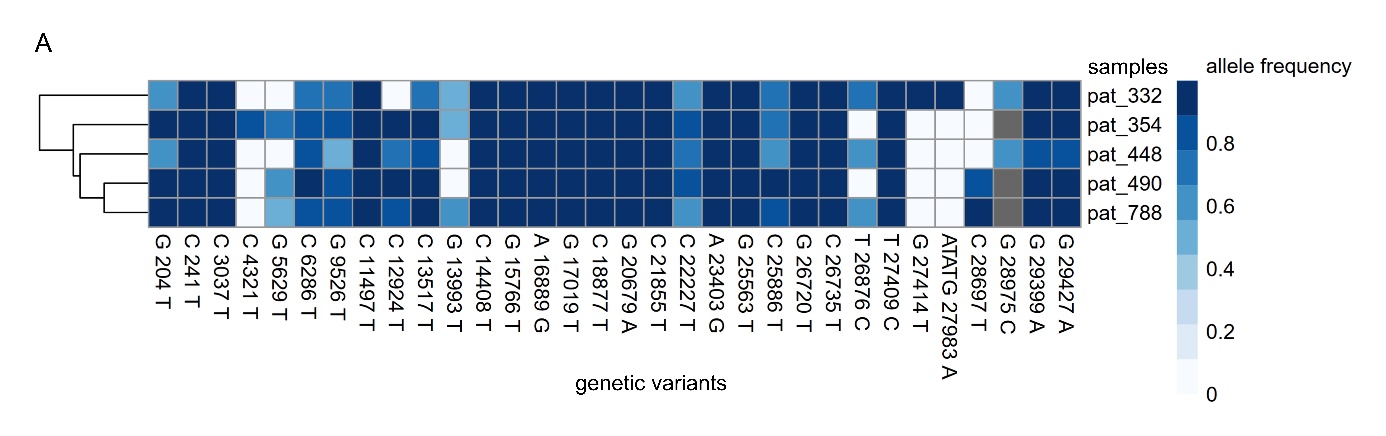

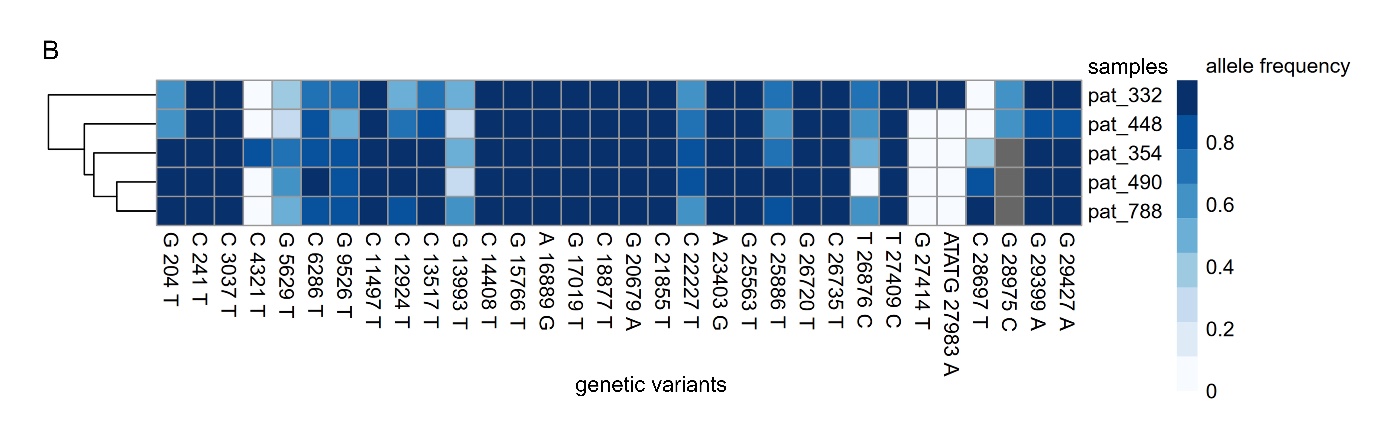

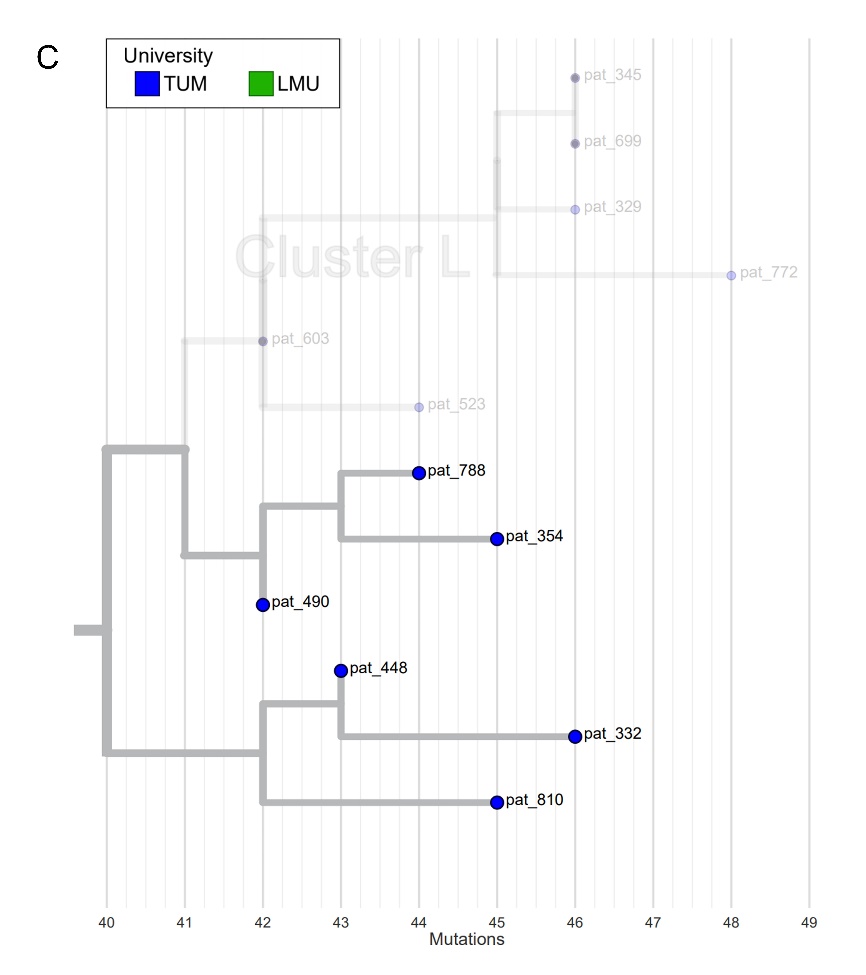

Suppl. Fig.20: Cluster S. (A) This cluster includes five samples from five probands, three staff members and two patients. The color gradient depicts allele frequencies, grey coloring denotes that the base call did not pass the quality control filters for a given variant. X-axes display genetic variants with allele frequencies >50% in at least one sample when compared to the reference genome (NC 045512.2) Each row denotes one sample included in the cluster. (B) Cluster S with low-frequency variants visible. (C) Representation of Cluster S in the phylogenetic tree. Five of the five samples were included in the tree.


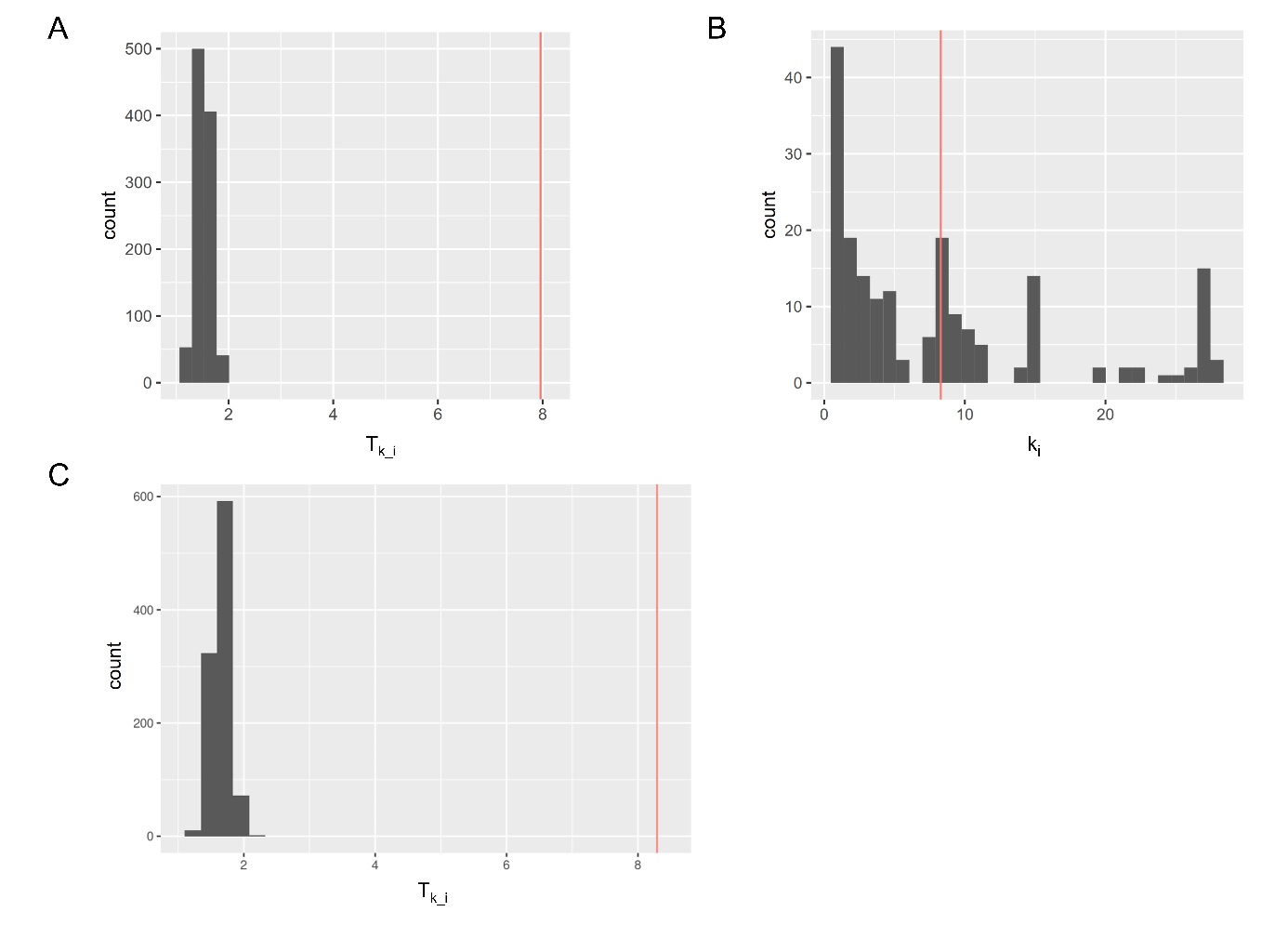


Suppl. Fig.21: Genetic relationship of TUM samples to LMU samples and each other
(A) Results of Monte Carlo Simulations with LMU samples using the last collected viral genome of an individual. X-axis displays the average number of viral genomes closely related to each TUM-individual (T_k_i_). The red line represents the average number of closely related samples in the TUM dataset (T_k_i_=7.96). TUM samples were significantly more closely related to each other than to the LMU samples (T_k_iLMU_=1.09-2.00). (1000 simulations, p=0.001).
(B) Close genetic relationships in TUM dataset. X-axis displays the number of viral genomes closely related to each TUM-individual (k_i_). The red line represents the average (T_k_i_=7.96).
(C) Results of Monte Carlo Simulations with LMU samples using the first collected viral genome of an individual. X-axis displays the average number of viral genomes closely related to each TUM-individual (T_k_i_). The red line represents the average number of closely related samples in the TUM dataset (T_k_i_=8.29). TUM samples were significantly more closely related to each other than to the LMU samples (T_k_iLMU_=1.19-2.08). (1000 simulations, p=0.001).


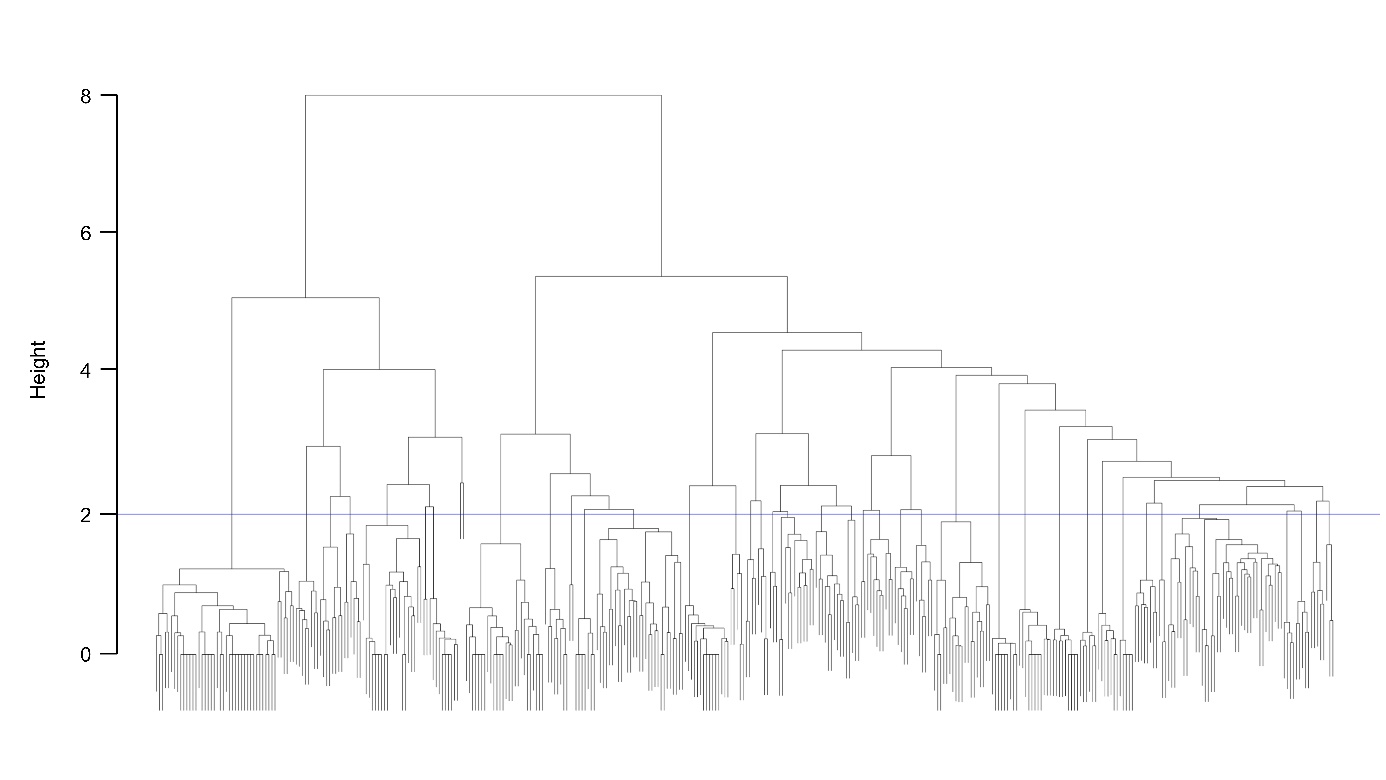
Suppl. Fig.22: Example of phylogenetic tree used during clustering analysis. The blue line represents the cutting height.

| **Genome coverage** | **Number of samples** |
| --- | --- |
| >80% <=90% | 45 |
| >90% <=95% | 71 |
| >95% | 503 |

Suppl. Fig.23: Genome coverage distribution of all 619 samples included in the analyses.

# Supplemental Table GISAID


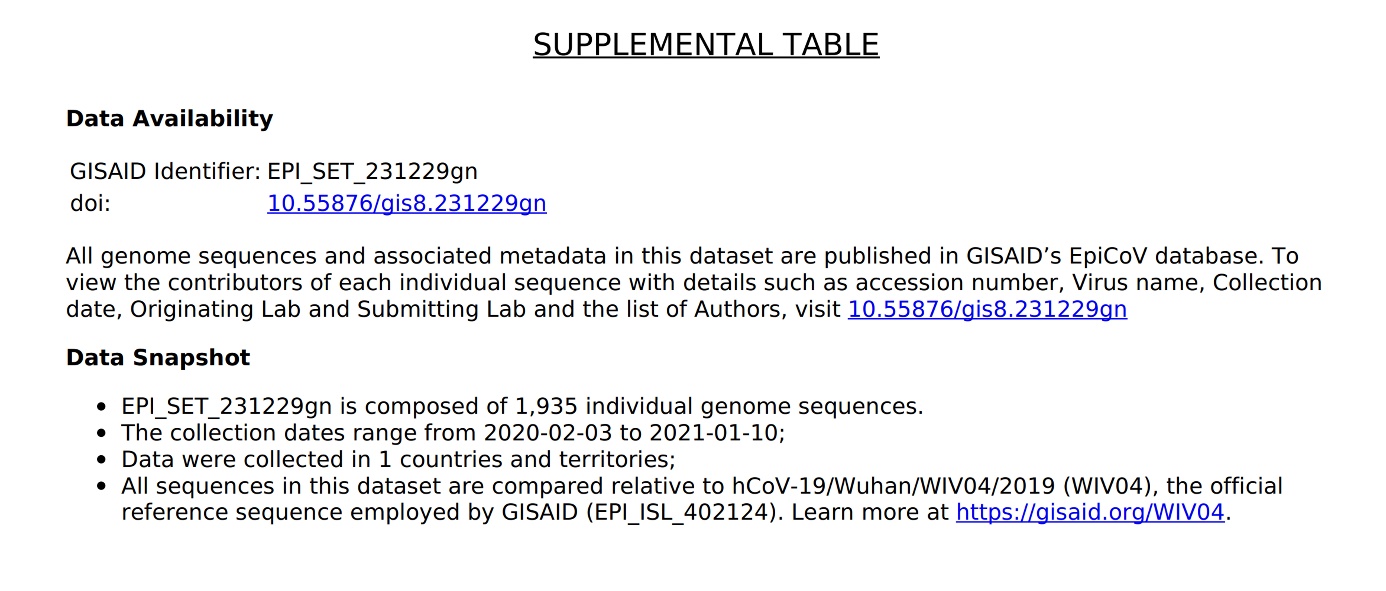


[1] ‘UG4001-04-CleanPlex-SARS-CoV-2-Panel-User-guide.pdf’. Accessed: Jun. 17, 2021. [Online]. Available: https://www.paragongenomics.com/wp-content/uploads/2021/02/UG4001-04-CleanPlex-SARS-CoV-2-Panel-User-guide.pdf

[2] Lusajo Mwakibete *et al.*, ‘ARTIC-NEB: SARS-CoV-2 Library PrepV.4’. protocols.io, Feb. 2021. [Online]. Available: https://www.protocols.io/view/artic-neb-sars-cov-2-library-prep-br77m9rn.pdf#page=1&zoom=auto,-23,848

[3] H. Li, ‘Aligning sequence reads, clone sequences and assembly contigs with BWA-MEM’, *arXiv e-prints*, p. arXiv:1303.3997, Mar. 2013.

[4] H. Li, ‘A statistical framework for SNP calling, mutation discovery, association mapping and population genetical parameter estimation from sequencing data’, *Bioinformatics*, vol. 27, no. 21, pp. 2987–2993, Nov. 2011, doi: 10.1093/bioinformatics/btr509.

[5] E. Garrison and G. Marth, ‘Haplotype-based variant detection from short-read sequencing’, *arXiv e-prints*, p. arXiv:1207.3907, Jul. 2012.

[6] J. Hadfield *et al.*, ‘Nextstrain: real-time tracking of pathogen evolution’, *Bioinformatics*, vol. 34, no. 23, pp. 4121–4123, Dec. 2018, doi: 10.1093/bioinformatics/bty407.

[7] P. V. Markov *et al.*, ‘The evolution of SARS-CoV-2’, *Nat Rev Microbiol*, vol. 21, no. 6, Art. no. 6, Jun. 2023, doi: 10.1038/s41579-023-00878-2.

[8] ‘Coronavirus disease (COVID-19)’. Accessed: Dec. 25, 2023. [Online]. Available: https://www.who.int/news-room/questions-and-answers/item/coronavirus-disease-covid-19

[9] S. A. Lauer *et al.*, ‘The Incubation Period of Coronavirus Disease 2019 (COVID-19) From Publicly Reported Confirmed Cases: Estimation and Application’, *Annals of Internal Medicine*, vol. 172, no. 9, pp. 577–582, May 2020, doi: 10.7326/M20-0504.
